# Antagonistic effects of selection on alleles associated with seed size and seed dormancy in wheat

**DOI:** 10.1186/s13059-025-03770-9

**Published:** 2025-09-25

**Authors:** Feilong Guo, Changbin Yin, Tian Li, Sitong Liu, Jiayu Dong, Hao Jiang, Yu Fang, Jun Wei, Yi Han, Yu Li, Hong Cao, Yuting Ning, Galal Khamis, Xin Deng, Ke Wang, Jirui Wang, Cuijun Zhang, Fei Lu, Yongxiu Liu

**Affiliations:** 1 State Key Laboratory of Forage Breeding-by-Design and Utilization, Institute of Botany, Chinese Academy of Sciences, Beijing, 100093 China; 2 University of Chinese Academy of Sciences, Beijing, 100049 China; 3 Key Laboratory of Plant Molecular Physiology, Institute of Botany, Chinese Academy of Sciences, Beijing, 100093 China; 4 Laboratory of Advanced Breeding Technologies, Institute of Genetics and Developmental Biology, Chinese Academy of Sciences, Beijing, 100101 China; 5 China National Botanical Garden, Beijing, 100093 China; 6 CAS-JIC Centre of Excellence for Plant and Microbial Science (CEPAMS), Institute of Genetics and Developmental Biology, Chinese Academy of Sciences, Beijing, 100101 China; 7 State Key Laboratory of Plant Diversity and Specialty Crops, Institute of Botany, Chinese Academy of Sciences, Beijing, 100093 China; 8 Institute of Crop Sciences, Chinese Academy of Agricultural Sciences, Beijing, 100081 China; 9 College of Plant Science and Technology, Beijing University of Agriculture, Beijing, 100096 China; 10 Triticeae Research Institute, Sichuan Agricultural University, Chengdu, 611130 China; 11 Department of Laser Applications in Metrology, Photochemistry and Agriculture, National Institute of Laser Enhanced Sciences (NILES), Cairo University, Giza, Egypt; 12 Shenzhen Branch, Guangdong Laboratory of Lingnan Modern Agriculture, Key Laboratory of Synthetic Biology, Ministry of Agriculture and Rural Affairs, Agricultural Genomics Institute at Shenzhen, Chinese Academy of Agricultural Sciences, Shenzhen, 518120 China

**Keywords:** Antagonistic effects, Genome-wide association study (GWAS), Seed dormancy, Seed size, Wheat

## Abstract

**Background:**

Seed dormancy and size are two crucial traits influencing crop yield, and they have undergone strong selection during cereal domestication and improvement. However, the genetic basis underlying the antagonistic effects between seed dormancy and seed size remains poorly understood.

**Results:**

Based on genome-wide association study, we perform a comprehensive comparative analysis of 545 global wheat accessions to dissect the genetic architecture of these two traits during wheat improvement. We detect a strong negative correlation between the accumulation of favorable alleles for seed dormancy and the accumulation of favorable alleles for seed size. At the wheat genome level, a set of SNPs harboring antagonistic alleles explain up to 26.56% and 47.21% of the phenotypic variation for seed dormancy and seed size, respectively. In contrast, a set of SNPs with synergistic alleles account for only 0.54% and 1.12% of the variation in both traits. During wheat breeding improvement, favorable alleles associated with increased seed size are preferentially selected, resulting in a compromise in seed dormancy. Under different climate conditions, the frequencies of haplotypes of the pleiotropic genes with antagonistic effects and synergistic loci collectively shape wheat diversity through balancing seed dormancy and seed size.

**Conclusions:**

Our findings reveal the genetic architecture underlying the observed weakening of seed dormancy as seed size increases during wheat improvement, enabling further genome-informed cultivar breeding to balance and improve seed dormancy and seed size traits.

**Supplementary Information:**

The online version contains supplementary material available at 10.1186/s13059-025-03770-9.

## Background

Seed dormancy and seed size are two critical functional traits, which are tightly related to plant environmental adaptation and crop yield [1, 2]. Seed dormancy controls the timing of germination in order for plants to complete their lifecycle in a favorable season. Seed size plays an important role in germination and seedling establishment by supplying nutrient substances. Previous studies have indicated a physiological and genetic association between seed dormancy and seed size. Larger seeds of *Dithyrea californica* and *Apium graveolens* exhibit lower dormant levels and faster germination compared to smaller seeds [3, 4]. In *Arabidopsis thaliana*, large seeds are associated with weaker dormancy among Iberian Peninsula accessions [5]. A comprehensive literature analysis revealed a significant correlation between seed size and germination rates across the majority of the examined 91 species, with 58 species exhibiting higher germination rates for larger seeds, while only 11 for smaller seeds [6]. However, recent research has shown that *Panicum halii* has evolved a genetic basis of antagonistic effects to produce either large seed/strong dormancy or small seed/weak dormancy trait combinations to adapt to xeric and mesic habitats, indicating the complex correlation between seed dormancy and seed size in nature ecosystems [7]. To the best of our knowledge, there is rare research on the genetic basis of antagonistic effects between seed dormancy and seed size in crops, which could be more intricate due to domestication and artificial selection.

Seed dormancy and seed size are complex traits influenced by numerous quantitative genetic loci (QTLs). Positional cloning of seed dormancy QTLs has successfully identified key genes that regulate seed dormancy, such as *DELLY OF GERMINATION1* (*DOG1*) in *A. thaliana* [8] and *Seed dormancy4* (*Sdr4*) and *Seed dormancy6* (*SD6*) in rice (*Oryza sativa*) [1, 9]. In wheat (*Triticum aestivum* L.), the gene *R-1* (*Tamyb10*), which controls both seed color and dormancy, is involved in the flavonoid synthesis pathway and ABA signal transduction [10, 11]. Furthermore, several major genes regulating seed dormancy have been identified in wheat, including *Mitogen-Activated Protein Kinase Kinase3* (*TaMKK3*) [12], *MOTHER OF FT AND TFL1* (*TaMFT-3A*/*TaPHS1*) [13], *ABI5 binding protein* (*TaAFP*) [14], and *Seed dormancy* (*TaSdr*) [15]. Regarding seed size/weight, thousand kernel weight (TKW) is the most stable and heritable characteristic mainly determined by seed size [2]. Several major genes, including *GRAIN WIDTH AND WEIGHT 2* (*TaGW2*) [16, 17], *UBIQUITIN RECEPTOR* (*TaDA1*) [18], *THOUSAND-GRAIN WEIGHT 7A* (*TaTGW-7A*) [19], *AUXIN/INDOLE ACETIC ACID REPRESSOR 21* (*TaIAA21*) [20], *POSITIVE REGULATOR OF GRAIN SIZE 1* (*TaPGS1*) [21], *CYTOCHROME P450* (*TaCYP78A5*) [22], *AWN LENGTH INHIBITOR 1* (*ALI-1*) [23], *AUXIN RESPONSE FACTOR* (*TaARF25*, *TaARF12*) [20, 24], and *PIN-FORMED 1* (*TaPIN1*) [25], have been reported to influence seed size and/or weight in wheat. In rice, *GRAIN LENGTH ON CHROMOSOME 7* (*OsGL7*) [26], *GRAIN LENGTH AND WEIGHT ON CHROMOSOME 7* (*OsGLW7/OsSPL13*) [27], *GRAIN SIZE 3* (*OsGS3*) [28], *DENSE AND ERECT PANICLE1* (*OsDEP1*) [29], *GRAIN WIDTH AND WEIGHT 2* (*OsGW2*) [30], and *GRAIN WIDTH ON CHROMOSOME 8* (*OsGW8/OsSPL16*) [31] have been demonstrated to regulate seed size and weight. In addition, dozens of seed size/weight-related genes have been cloned in maize (*Zea mays*), barley (*Hordeum vulgare*), and other cereal crops [32, 33]. Although the genetics of seed size and dormancy have been extensively studied respectively, limited studies have focused on the genetic architecture and antagonistic effects between these two traits in crops.

In this study, we conducted a large-scale GWAS based on 60 million filtered SNPs to dissect the relationship and genetic basis of seed dormancy and seed size during wheat breeding. Both seed dormancy and seed size traits exhibited relatively high heritability. Furthermore, seed dormancy showed significant phenotypic and genetic correlations with seed size trait. A strong negative correlation was observed between the accumulation of favorable alleles for seed dormancy and those for seed size. In addition, we discovered that 18.59–28.60% of marker-trait associations (MTA) SNPs and 13.61–32.39% of lead SNPs (the peak SNP in GWAS signal regions) were characterized by alleles with antagonistic effects on these two traits, a proportion much higher than that of SNPs harboring alleles with synergistic effects. This further indicates a strong negative correlations between the two traits. Two major genes, *Tamyb10* and *TaGW2*, exhibit pleiotropic effects on seed dormancy and seed size with opposing influences. Moreover, three novel synergistic loci affecting both traits were identified. A candidate gene *RNA-binding protein* (*TaRBP-4A*) was identified for the synergistic locus *Qgd-gs.4A.1*, which may improve both seed dormancy and seed size traits. Our findings provide insights into the impacts of breeding selection on the seed dormancy and seed size in common wheat. We also generated extensive allelic composition data related to seed dormancy and seed size for each of the 545 wheat accessions, which will be beneficial for simultaneous optimization of these two key agronomic traits in future breeding programs.

## Results

### Population structure of 545 wheat accessions

**Fig. 1:**
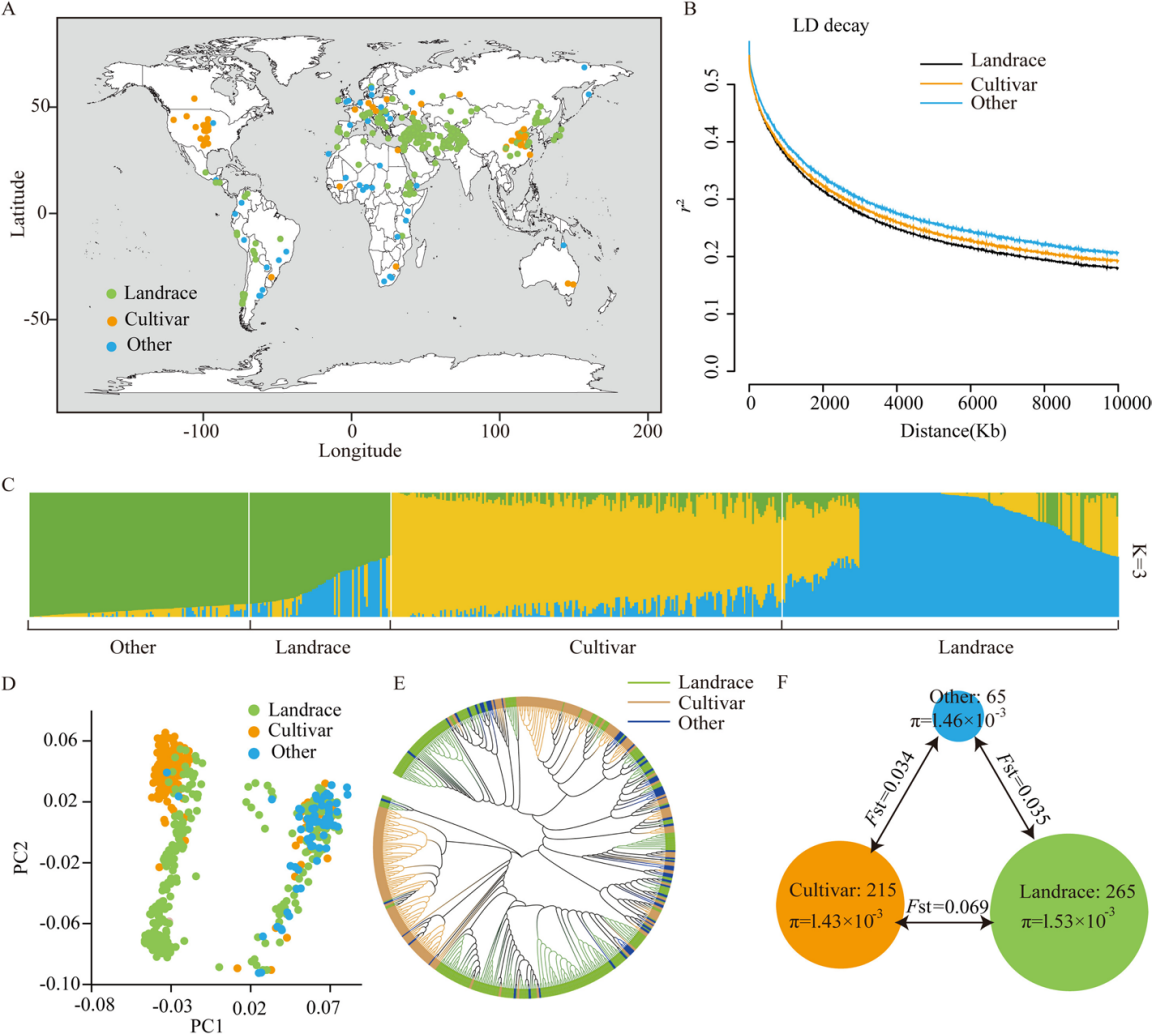
Geographic distribution, linkage disequilibrium decay, population structure, and genetic diversity of 545 wheat accessions.** A** Geographic distribution of the 545 wheat accessions. Different dots represent collections from different geographic places. Green dots represent landraces, orange dots represent cultivars, and blue dots represent other wheat accessions. **B** Decay of linkage disequilibrium (LD) in wheat genome. Black line represents landrace, orange line represents cultivar, and blue line represents other wheat accession.** C** Population structure analysis of the 545 common wheat accessions inferred with *K* = 3. **D** Principal components analysis (PCA) plots of the first two principle components (PC1 and PC2) for 545 wheat accessions. **E** Phylogenetic tree of 545 common wheat accessions constructed using whole-genome SNPs. Green represents landrace, orange represents cultivar, and blue represents other wheat accessions. **F** Statistics for genetic diversity and population differentiation. The size of the circles represents the number of accessions and nucleotide diversity (π) within the corresponding subpopulation. The values between pairs of subpopulations indicate the population divergence (*F*st)

In this study, 545 hexaploid wheat accessions originated from 76 countries, including 265 traditional local wheat accessions (landraces), 215 advanced common wheat accessions (cultivars), and 65 other common wheat breeding material (others), were selected to investigate seed dormancy and seed size (Fig. 1A, Additional file 1: Table S1). Moreover, high-coverage whole-genome sequencing (~ 10 ×) enabled the identification of a total of 63,379,140 filtered SNPs with minor allele frequency (MAF) > 0.05 across 545 wheat accessions (Additional file 2: Fig. S1).

We calculated the decay rate of linkage disequilibrium (LD) as the pairwise correlation coefficient (*r*^2^) from the maximum value to the half-maximum, which reached 3.01 Mb, 3.48 Mb, and 3.35 Mb for landraces, cultivars, and other wheat accessions, respectively (Fig. 1B). To explore population differentiation among worldwide wheat accessions, a population structure analysis was constructed (Fig. 1C), along with principle component analysis (PCA) (Fig. 1D) and phylogenetic tree construction (Fig. 1E). The population structure analysis revealed that most landraces and cultivars were separated from other accessions at *K* = 3 (Fig. 1C). PCA also demonstrated that most landrace accessions could be distinguished from cultivars by PC1 and PC2 (Fig. 1D), although a few accessions exhibited proximity to other wheat accessions. These findings were consistent with the corresponding phylogenetic tree (Fig. 1E). Furthermore, we performed a comprehensive comparison of genetic diversity and differentiation across populations. The nucleotide diversity (π) was lower in cultivars (π_cultivar_ = 1.43 × 10^−3^) compared to landraces (π_landrace_ = 1.53 × 10^−3^) and other wheat accessions (π_other_ = 1.46 × 10^−3^) (Fig. 1F). Additionally, the fixation index (*F*st) values, which describe the genetic differentiation level between two populations, were 0.069 for cultivar vs. landrace, 0.035 for other vs. landrace, and 0.034 for other vs. cultivar (Fig. 1F).

### Seed dormancy and seed size traits of 545 wheat accessions

The dormancy and size traits of wheat seeds were analyzed in four environments, namely ZX2021, ZX2022, ZX2023, and BJ2023. The percentage of germinated seeds after 3 days (GP3D/%) and 7 days (GP7D/%) imbibition, as well as thousand kernel weight (TKW/g), kernel length (KL/mm), and kernel width (KW/mm), were determined (Additional file 1: Table S2). To eliminate any bias resulting from environmental effects (phenotype-location/year effect), the best linear unbiased estimator (BLUE) values for GP3D-BLUE, GP7D-BLUE, TKW-BLUE, KL-BLUE, and KW-BLUE were also calculated, ranging from 6.14 to 95.23%, 12.43 to 97.69%, 20.11 to 61.73 g, 4.58 to 7.82 mm, and 2.38 to 3.62 mm, respectively (Additional file 1: Table S3). Analysis of variance (ANOVA) revealed strong genetic effects for GP3D, GP7D, TKW, KL, and KW, which were consistent with their relatively high broad-sense heritability (*H*^2^ 0.8517–0.9598, Additional file 1: Table S3). These heritability estimates are higher than previous study [34], likely due to the phenotypic measurements spanning three years (2021, 2022, and 2023), two locations (Zhaoxian and Beijing), and two replicates.

**Fig. 2:**
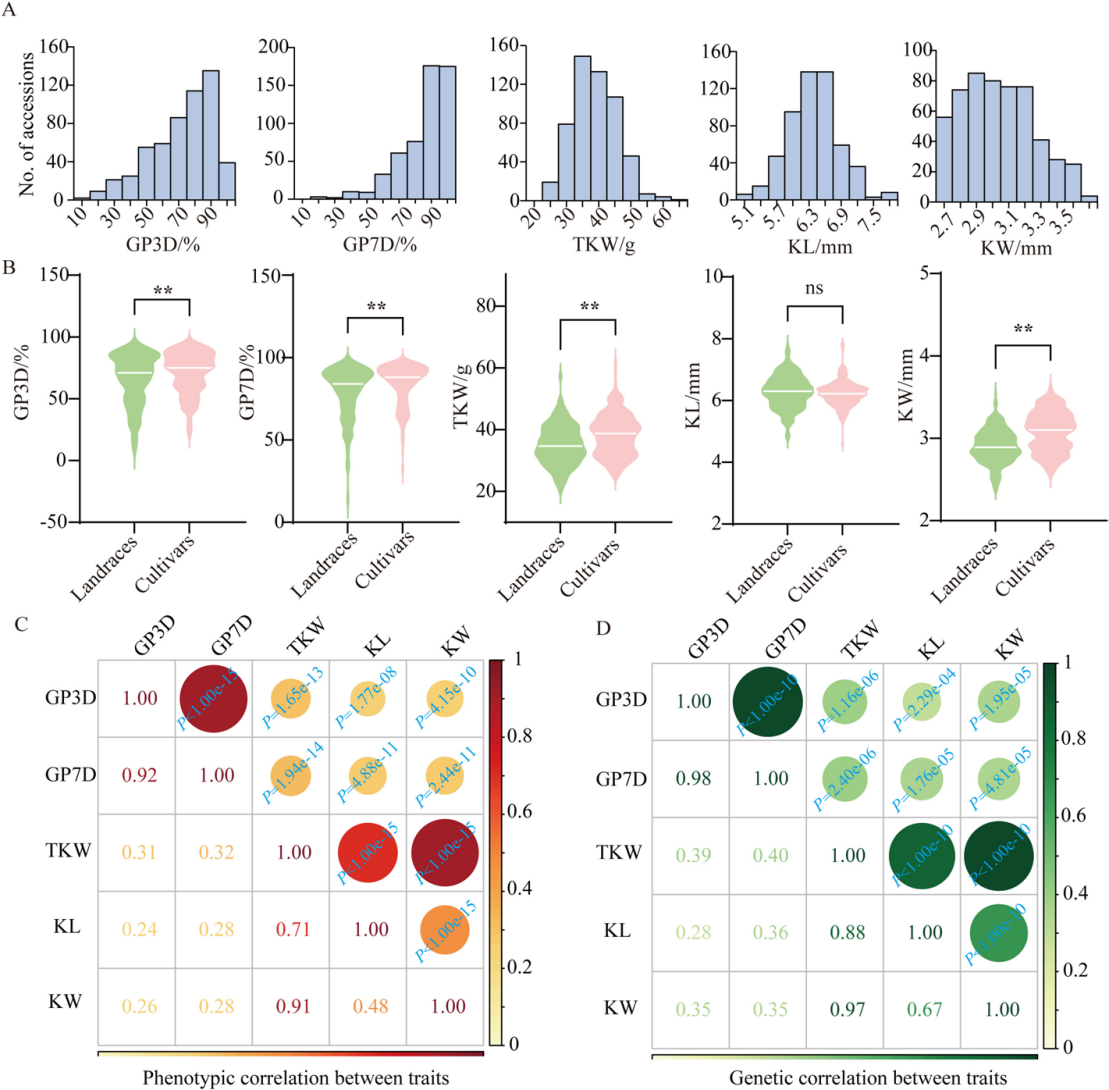
Phenotypic analyses of 545 wheat accessions.** A** Frequency distribution of GP3D, GP7D, TKW, KL, and KW values. **B** Comparison of landraces and cultivars for GP3D, GP7D, TKW, KL, and KW values. Significant differences were determined using the Student’s *t*-test. *, 0.01 < *P* < 0.05; **, *P* < 0.01. **C**–**D** The phenotypic (C) and genetic (D) correlations between traits for GP3D, GP7D, TKW, KL, and KW

Correlation analysis showed that GP3D-BLUE, GP7D-BLUE, TKW-BLUE, KL-BLUE, and KW-BLUE were highly correlated with phenotypes across the environments (Additional file 2: Fig. S2A–E). Frequency histograms of GP for 545 worldwide wheat accessions showed that most wheat accessions exhibited low dormancy and high GP (≥ 90%) (Fig. 2A). Notably, the GP3D-BLUE, GP7D-BLUE, TKW-BLUE, and KW-BLUE values for wheat landraces were significantly lower than those for cultivars (Fig. 2B). Furthermore, there has been a rapid increase in GP, TKW, KL, and KW over the past 120 years during wheat breeding in cultivars (Additional file 2: Fig. S2F). Additionally, seed dormancy traits were significantly correlated with seed size traits, especially GP7D and TKW (Fig. 2C). Consistent with these findings, bivariate genome-based restricted maximum likelihood (GREML) analyses revealed significant positive genetic correlations between seed germination and seed size (*r*_g_ = 0.28–0.40) (Fig. 2D).

### Identification of genomic loci controlling seed dormancy and seed size by GWAS

**Fig. 3:**
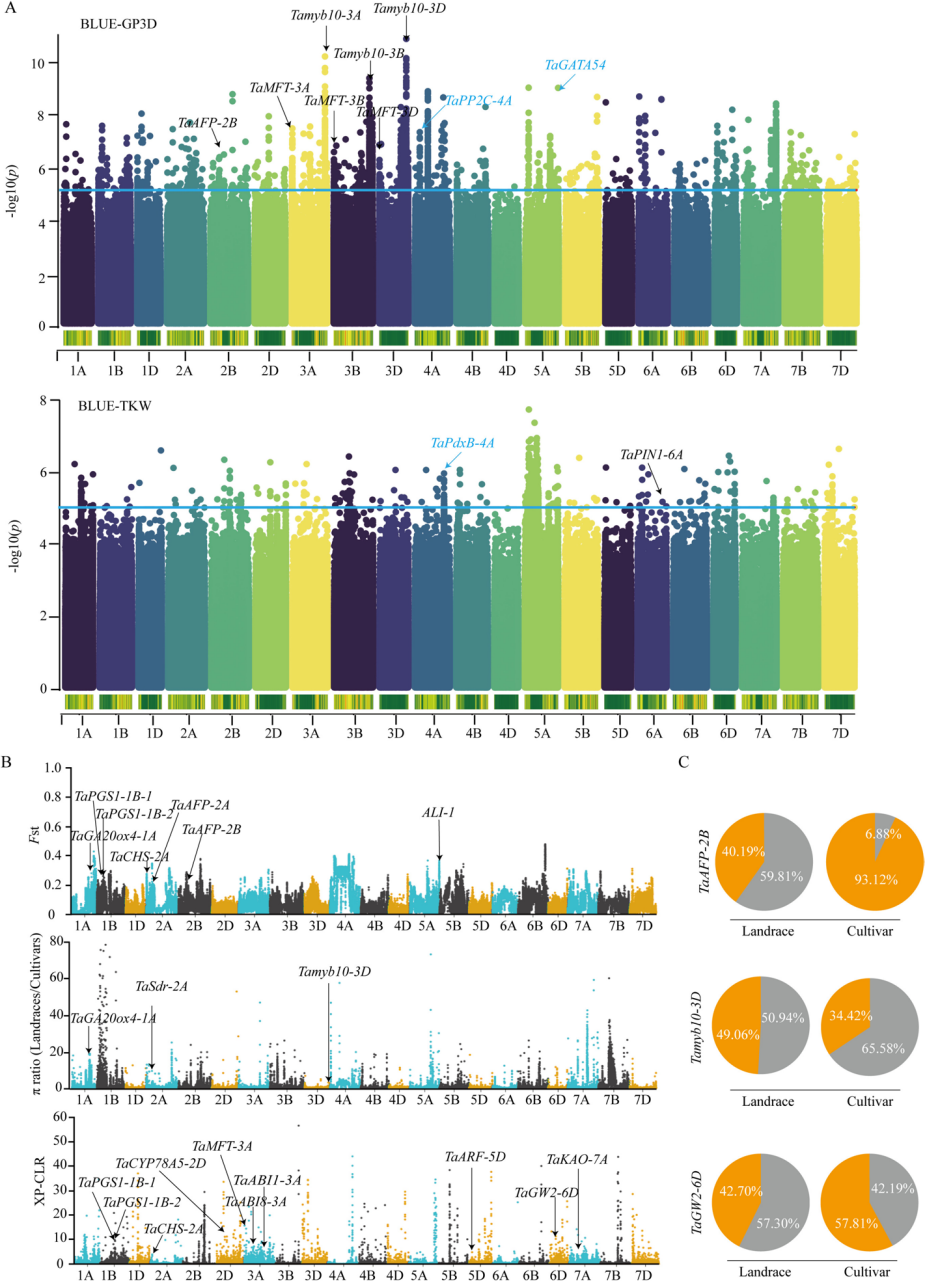
Profiling of genome-wide association analysis, genome-specific selection signatures, and sweeps.** A** Manhattan plots showing SNP marker-trait associations for the BLUE-GP3D and BLUE-TKW. Black arrows indicate known seed dormancy/size genes, and blue arrows indicate candidate seed dormancy/size genes. Blue line represents the significance threshold (− log_10_ [*P*-value] > 5).** B** Selection signatures and sweeps were identified using *F*st, π ratio (π_Landrace_/π_Cultivar_) and XP-CLR. The genome-wide threshold was defined as the top 5% of values. The known seed dormancy genes and seed size-related genes in the selective regions are labeled with black arrows. **C** The comparison of two major haplotype frequencies between landraces and cultivars for seed dormancy/size genes targeted by selection. The haplotype with higher frequency in landrace is designated as Hap 1 (gray) and the one with lower frequency is designated as Hap 2 (orange)

To elucidate the genetic basis of seed dormancy and seed size during wheat breeding, we performed GWAS for the phenotypic values of the GP3D, GP7D, TKW, KL, and KW in each of the environments and their BLUE values using 63,379,140 SNPs (Fig. 3A, Additional file 2: Fig. S3). Significant SNPs associated with traits (− log_10_ [*P*-value] > 5) were identified and the union was taken across all environments: 8747 for GP3D, 18,055 for GP7D, 2680 for TKW, 1130 for KL, and 2721 for KW (Additional file 1: Table S4–S8). Among these significant MTA loci, known genes associated with seed dormancy such as *Tamyb10-3A/B/D* [10, 11], *TaMFT-3A/B/D* [13], *TaAFP-2B* [14], *TaSdr-2D* [15], and *TaMKK3* [12], as well as seed size-related genes such as *TaPGS-1D* [21], *TaARF12-2A* [24], *TaPIN1-6A* [25], *TaPRR1-B1* [35], *KAT-2B* [36], and *TaAGL6-B* [37], other known QTLs [38–62], and genes orthologous to rice gene [63–74] were identified (Additional file 1: Table S9). A total of 632 loci were identified across all environments within the GWAS analysis. Among these loci, new associations were detected in more than two environments. Specifically, there were 37 new loci associated with GP3D, 60 with GP7D, 7 with TKW, 6 with KL, and 14 with KW (Additional file 1: Table S9).

Two novel candidate genes associated with seed dormancy, *Protein phosphatase 2C* (*TaPP2C-4A*, *TraesCS4A02G094300*) and *GATA* transcription factor (*TaGATA54*, *TraesCS5A02G538600*), were identified based on GWAS and RNA-seq analysis (Additional file 2: Fig. S4). The expressions of *TaPP2C-4A* and *TaGATA54* exhibited significant differences between dormant seeds and dormancy-released seeds, as well as different dormancy degree wheat cultivars (Additional file 2: Fig. S4B and S4E). The orthologue of *TaPP2C-4A* in rice is *OsPP2C30* (74.04% identity), which acts an ABA signal receptor during seed germination and early seedling growth [75]. Three major haplotypes at *TaPP2C-4A* were identified among 545 wheat accessions (Additional file 1: Table S10), and their effects were determined with Hap1 > Hap2 > Hap3 on seed dormancy (Additional file 2: Fig. S4C). Similarly, three major haplotypes of *TaGATA54* were found among the same set of wheat accessions (Additional file 1: Table S10), and they showed associations with seed dormancy as well (Additional file 2: Fig. S4F). Previous research has suggested that *TaGATA54* may negatively regulate seed dormancy in wheat based on the changes of expression levels between weak and strong dormancy wheat varieties [76]. In addition, based on RNA-seq analysis and haplotypes analysis, *Erythronate-4-phosphate dehydrogenase* (*TaPdxB-4A*, *TraesCS4A02G340400*) was also implicated in regulating seed size in wheat (Additional file 1: Table S10, Additional file 2: Fig. S5). RNA-seq results showed that the *TaPdxB-4A* was significantly highly expressed in the developing seeds (Additional file 2: Fig. S5B). Haplotypes analysis result showed that the TKW and KW of Hap 2/3 were significantly higher than Hap 1 (Additional file 2: Fig. S5C).

### Genome-wide selection signatures and sweeps of seed dormancy and seed size

To confirm whether the loci identified by GWAS have been under selection during wheat improvement, breeding selection signatures and sweeps were detected between wheat landrace and cultivar populations using the π ratio (π_Landrace_/π_Cultivar_), *F*st and cross-population composite likelihood ratio (XP-CLR) scores. Each of these analyses indicated that a wide variety of loci have been targeted during wheat breeding improvement (Fig. 3B). In total, 1870 Mb, representing approximately 13.30% of the wheat genome, was shown to be affected by putative selective sweeps with at least one method, including 988.6 Mb on the A sub-genome (5476 genes), 719.5 Mb on the B sub-genome (3652 genes), and 252.4 Mb on the D sub-genome (2255 genes) (Additional file 1: Table S11). By comparing the ratio of selected genes per Mb region, we found that the A (1.11 genes/Mb) and B sub-genomes (0.70 genes/Mb) were subject to be stronger breeding selection than the D sub-genome (0.57 genes/Mb) (Additional file 1: Table S11). Among all the triplets of genes in the common wheat genome, 1335 genes showed a selection signal in the A sub-genome, 1536 in the B sub-genome, and 1610 in the D sub-genome (Additional file 1: Table S11–S12). By integrating GWAS results with selection signatures and sweeps analysis, it was found that 39.40% of the loci (249 of 632) were significantly selected during wheat improvement (Additional file 1: Table S9). Additionally, a comparable analysis of major haplotypes between landrace and cultivar further confirmed the selection in the accumulation of these genes (Fig. 3C, Additional file 2: Fig. S6), such as seed dormancy-related genes *TaGA20ox4-1A* [77], *TaSdr-2A* [15], *TaAFP-2A/B* [14], *TaMFT-3A* [13], *TaABI1-3A* [77], *TaABI8-3A* [77], *Tamyb10-3D* [10], and *TaKAO-7A* [78], as well as genes related to seed size *TaPGS1-1B-1/2* [21], *TaCYP78A5-2D* [22], *ALI-1* [23], *TaARF25-5D* [20], and *TaGW2-6D* [17].

### Changes in favorable allele frequency (FAF) for seed dormancy and seed size

**Fig. 4:**
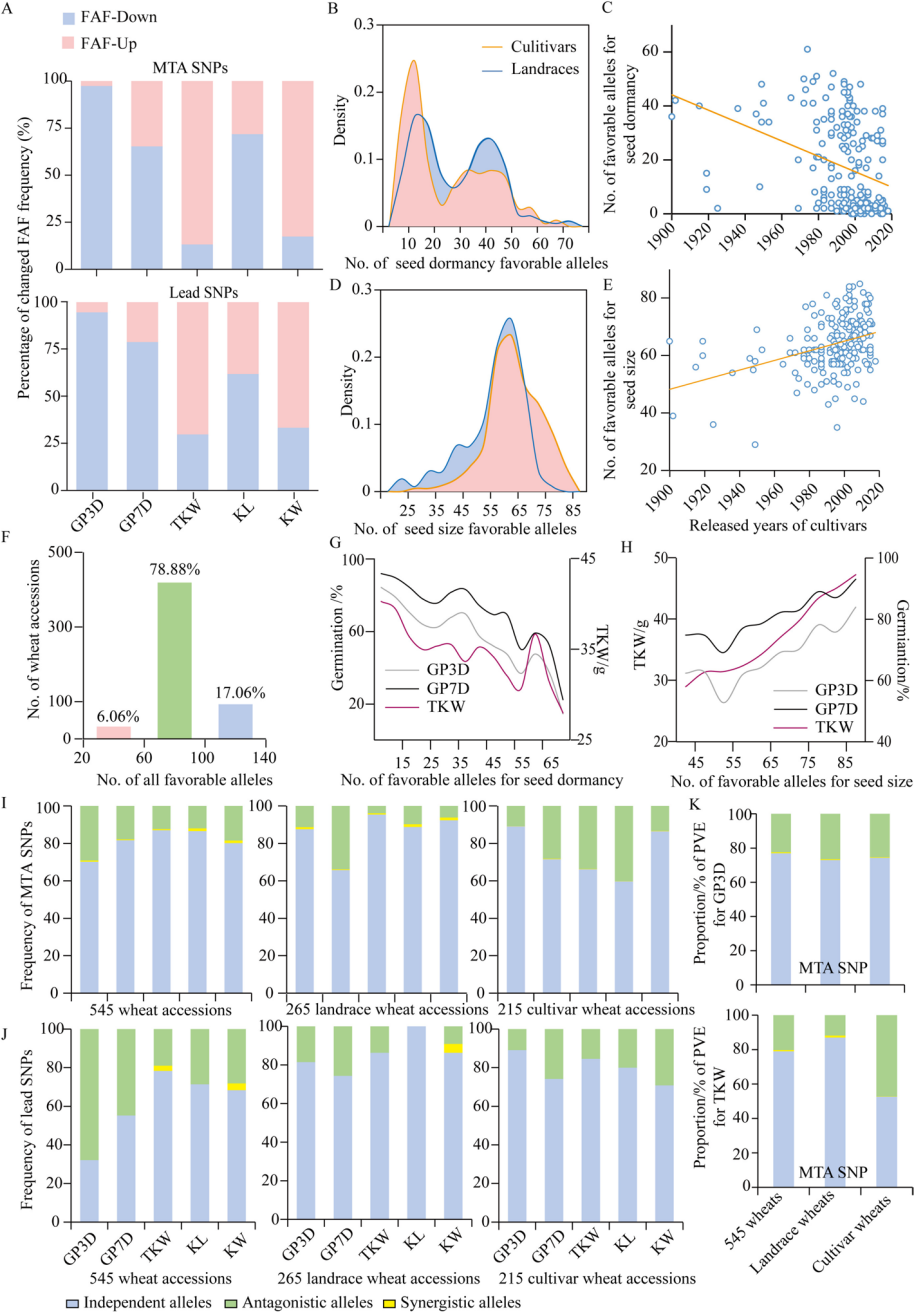
Changes of FAF and the correlation between seed dormancy and seed size during modern wheat breeding improvement.** A** Percentages of MTAs (upper panel) and lead SNPs (lower panel) with increased or decreased FAF. FAF-Up and FAF-Down respectively represent the proportion of SNPs exhibiting increased or decreased FAF in comparison between cultivars and landraces.** B** Density distribution of favorable alleles for seed dormancy between landraces and cultivars. The pink line represents wheat cultivars, and blue line represents wheat landraces. **C** The number changes of favorable alleles for seed dormancy across different released years for cultivars. **D** Density distribution of favorable alleles for seed size between landraces and cultivars. The pink line represents wheat cultivars, and blue line represents wheat landraces.** E** The number changes of favorable alleles for seed size across different released years for cultivars. **F** The number of wheat accessions possessing favorable alleles for seed dormancy and seed size. Favorable alleles for seed dormancy and seed size in each wheat accession were accumulated and classified into three categories based on the total number of favorable allele (ranging from 20 to 60, from 60 to 100, and from 100 to 140). **G** Change trend plots indicating the negative correlation between the number of favorable alleles for seed dormancy and seed size trait. The gray, black, and purple lines represent GP3D, GP7D, and TKW, respectively.** H** Change trend plots indicating the positive correlation between the number of favorable alleles of seed size and germination (GP3D). **I**–**J** Frequency of SNPs harboring alleles with synergistic, antagonistic, and independent effects among MTA SNPs (**I**) and lead SNPs (**J**) across 545 wheat accessions, 265 landrace accessions, and 215 cultivar accessions. The blue, green, and yellow boxes represent SNPs harboring alleles with independent effects (independent alleles), antagonistic effects (antagonistic alleles), and synergistic effects (synergistic alleles). The *t*-test was used to determine differences in the effects of MTA or lead SNPs on seed size or seed dormancy.** K** Proportion of PVE for GP3D (upper panel) and TKW (lower panel) based on MTA SNPs in all wheat accessions, landrace accessions, and cultivar accessions. PVE indicating phenotypic variation explained

To investigate the effects of wheat breeding selection on bi-allelic variants significantly associated with seed dormancy and seed size, we compared the FAF between the landrace and the cultivar. We also generated a comprehensive allelic composition for each of the 545 wheat accessions at lead SNP markers of GP, TKW, KL, and KW (Additional file 2: Fig. S7A). Favorable alleles were defined as SNPs associated with higher TKW, KL, and KW for seed size traits, reduced GP for seed dormancy traits. Through direct analysis of all GWAS hits of BLUE (− log_10_ [*P*-value] > 5), we discovered that 91.59% and 54.82% of MTA SNPs for GP3D and GP7D, respectively, showed decreased FAF in cultivars compared to landraces. Additionally, 86.82%, 28.19%, and 82.61% of MTA SNPs for TKW, KL, and KW, respectively, exhibited increased FAF in cultivars (Fig. 4A, Additional file 1: Table S13, Additional file 2: Fig. S7B). Similar results were obtained by analyzing of GWAS-associated lead SNPs, which showed decreased FAF for seed dormancy and increased FAF for seed size (Fig. 4A, Additional file 1: Table S13, Additional file 2: Fig. S7C).

Based on the allelic composition of each cultivar and landrace at lead SNPs, our findings revealed a significant decrease in the proportions of favorable alleles for seed dormancy in cultivar populations (Fig. 4B). Furthermore, an analysis of the number of favorable alleles in cultivars from different breeding periods confirmed a rapid decline in favorable alleles (Fig. 4C). Additionally, there was a tendency for average values of GP3D (*R*^2^ = 0.92) and GP7D (*R*^2^ = 0.83) to decrease with increasing number of favorable alleles (Additional file 2: Fig. S8A). Conversely, we observed a substantial increase in the proportions of favorable alleles in the cultivars compared to landraces for seed size traits (Fig. 4D), with a rapid increase over time in released cultivars as well (Fig. 4E). Moreover, there was also a tendency for average values of TKW (*R*^2^ = 0.87), KL (*R*^2^ = 0.94), and KW (*R*^2^ = 0.92) to increase with the increasing number of favorable alleles (Additional file 2: Fig. S8B). Meanwhile, only 17.06% of the wheat accessions analyzed carried more than 100 favorable alleles (Fig. 4F, Additional file 1: Table S14).

### Negative correlation between seed dormancy and seed size in wheat

As there were overlapping seed dormancy QTLs and known seed size QTLs [46, 47, 54, 56, 57, 59, 61, 79–93], as well as seed size QTLs and known seed dormancy QTLs [41, 44, 94, 95] (Additional file 1: Table S15), we further analyzed whether there was a significant correlation between seed dormancy and seed size. As the number of favorable alleles associated with seed dormancy traits increased, the seed size (TKW, KL, and KW) and germination (GP3D and GP7D) tended to decrease (Fig. 4G, Additional file 2: Fig. S8C). Conversely, as the number of favorable alleles associated with seed size traits increased, germination (GP3D and GP7D) and seed size (TKW, KL, and KW) also tended to increase (Fig. 4H, Additional file 2: Fig. S8D).

Moreover, we further analyzed 18 known seed dormancy genes and 18 known seed size genes (Additional file 2: Fig. S9–S10), including those identified in the sweep region, GWAS, and other known major genes reported in previous studies. Haplotype analysis revealed that the favorable haplotype with low GP potentially tended to have low TKW for most of the seed dormancy genes (10 out of 18 genes), including *TaABI8-3A*, *TaMFT-3A/B/D*, *Tamyb10-3A*/*B*/*D*, *TaMKK3-5D*, and *TaSD6-7A/B*. Conversely, the favorable haplotypes of *TaAFP-2B* exhibited low GP and high TKW (Additional file 1: Table S16, Additional file 2: Fig. S9). For seed size-related genes, the favorable haplotypes for high TKW tended to have high GP for most of seed size genes (11 out of 18 genes), including *TaPGS1-1B-1/2*, *TaARF25-5D*, *TaGW2-6A/D*, *TaARF12-2A*, *TaPGS1-1D*, *TaPIN1-6A*, *TaPRR1-B1*, *TaSPL17-7A*, and *TaIAA21-B*, while the favorable haplotypes for *TaDA1-2B* showed large seed and low GP (Additional file 1: Table S17, Additional file 2: Fig. S10). Consistent with favorable alleles, a similar change trend was observed through favorable haplotype association analysis of these seed dormancy and seed size genes (Additional file 2: Fig. S11A–B). The number of favorable haplotypes for seed size genes rapidly increased in released wheat cultivars over time, while the number of favorable haplotypes for seed dormancy genes rapidly decreased in released wheat cultivars (Additional file 2: Fig. S11C–D).

To further elucidate the correlation between seed dormancy and seed size at the genome level, we analyzed all MTA SNPs and lead SNPs related to seed dormancy and seed size based on BLUE-GWAS results using 545 wheat accessions, 265 landrace wheat accessions, and 215 cultivar wheat accessions, respectively (Additional file 2: Fig. S12). Among these MTA SNPs, 71.20–80.73% harbored alleles with independent effects (affecting only one trait), 0.21–0.76% harbored alleles with synergistic effects (high TKW and low GP), and 18.59–28.60% harbored alleles with antagonistic effects (high TKW with high GP or low TKW with low GP) (Fig. 4I, Additional file 1: Table S18). Notably, the proportion of SNPs with synergistic alleles was significantly higher in landraces (~0.76%) than in cultivars (~0.21%). Conversely, the proportion of SNPs with antagonistic alleles was significantly higher than in cultivars (~28.60%) than in landraces (~24.90%) (Fig. 4I, Additional file 1: Table S18). Meanwhile, the set of SNPs harboring antagonistic alleles explained 22.49–26.56% and 11.82–47.21% of the phenotypic variation for GP3D and TKW. In contrast, the set of SNPs with synergistic alleles only explained 0.36–0.54% and 0.13–1.12% of the phenotypic variation for GP3D and TKW, respectively (Fig. 4K, Additional file 1: Table S19). Similar results were also obtained by analyzing lead SNPs (Fig. 4J, Additional file 1: Table S18).

### Overlapping regions governing seed dormancy and seed size reveal antagonistic effects during wheat breeding

**Fig. 5:**
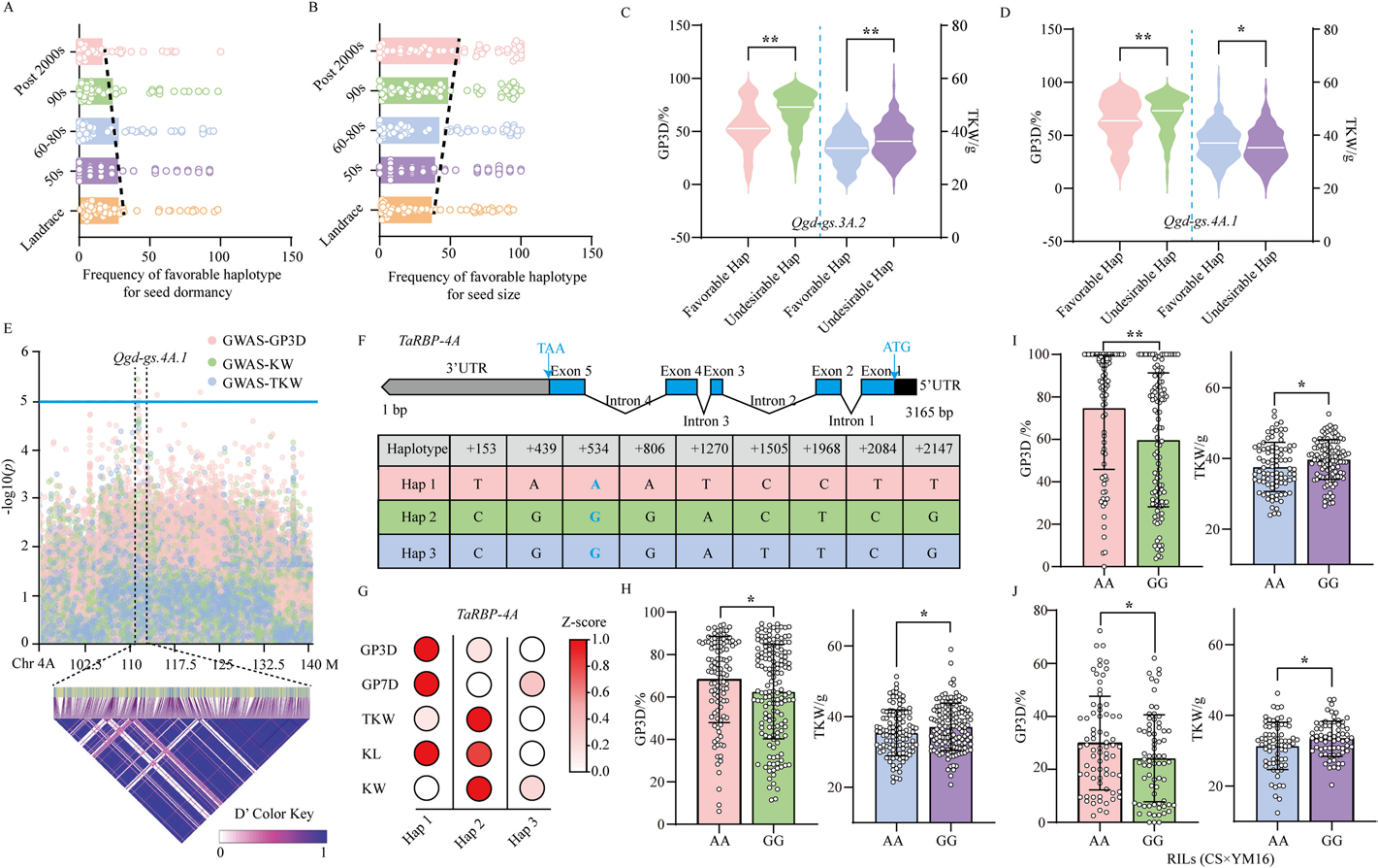
Synergistic effect analysis for *TaRBP-4A*. **A**–**B** Changes in the number of favorable haplotypes in the antagonistic regions for seed dormancy (**A**) and seed size (**B**) from landraces to released cultivars across different breeding periods. The bar plot represents the average frequency of favorable haplotypes for seed dormancy or seed size. The tagSNPs from overlapping region were extracted and used to classify the haplotypes. **C**–**D** Differences in GP3D and TKW between favorable and undesirable haplotype for *Qgd-gs.3A.2* and *Qgd-gs.4A.1*. **E** Manhattan plots and pairwise LD analysis showing associations between SNP markers and seed dormancy or seed size traits on chromosome 4A. Blue line indicates the significance threshold (− log_10_ [*P*-value] > 5). **F** Gene structure and the haplotypes of *TaRBP-4A*. The blue highlight indicates that CAPS markers are developed based on this SNP.** G** Association of seed phenotypes with *TaRBP-4A* haplotypes. The color-coded scale shows normalized phenotype values. **H**–**J** Utilizing CAPS Markers, comparisons were conducted on 260 randomly selected wheat accessions from the current wheat population (**H**), 198 other wheat accessions (**I**), and 147 RILs (**J**) derived from Chinese spring (CS) × Yangmai16 (YM16) cross to assess the variations in GP3D and TKW associated with the AA and GG alleles. Dots represent phenotypic values for each wheat accession. Significant differences were determined using the Student’s *t*-test. *, 0.01 < *P* < 0.05; **, *P* < 0.01

A total of 66 regions associated with seed dormancy were found to overlap with regions linked to seed size based on the BLUE-GWAS hits and GWAS hits identified in at least two environments for all wheat accessions, as well as those for 265 landrace wheat accessions and 215 cultivar wheat accessions (Additional file 2: Fig. S13). Furthermore, tagSNPs of 66 overlapping regions were extracted for haplotype block analysis (Additional file 1: Table S20). The haplotype analysis revealed that almost all of the haplotypes in the overlapping regions exhibited an antagonistic phenotype, i.e., high TKW with high GP or low TKW with low GP, and 21 overlapping regions showed a significant difference between the favorable haplotype and the undesirable haplotype (Additional file 1: Table S21). During wheat breeding period, the strong selection led to the fixation of favorable alleles for seed size rather than seed dormancy in these antagonistic linkage regions (Fig. 5A–B). Among them, *Qgd-gs.3A.2* with significantly antagonistic effect harbored a known seed dormancy gene *Tamyb10-3A* (Fig. 5C). Three regions (*Qgd-gs.4A.1*, *Qgd-gs.5D.2*, and *Qgd-gs.7A.1*) were found to show significant difference between favorable haplotype and the undesirable haplotype with the synergistic effects (Fig. 5D, Additional file 1: Table S21, Additional file 2: Fig. S14). In addition, 29 regions were found to overlap with selective sweep hits, and 24 genes orthologous to rice genes [21, 96–118] were identified as being involved in regulating seed size or germination within these regions (Additional file 1: Table S22).

Especially, one *RNA-binding protein* gene (*TaRBP-4A*, *TraesCS4A02G099300*) was identified based on GWAS and RNA-seq analysis from synergistic region *Qgd-gs.4A.1*, which positively enhanced seed dormancy and seed size in wheat (Fig. 5E, Additional file 2: Fig. S15A). Three major haplotypes at *TaRBP-4A* were identified among 545 wheat accessions (Fig. 5F, Additional file 1: Table S10). Significant differences were observed in seed dormancy and seed size among three haplotypes (Fig. 5G, Additional file 2: Fig. S15B). The CAPS marker was developed based on the A/G SNP located at 534 bp of *TaRBP-4A* gene sequence. This marker demonstrated a significant association with GP3D and TKW in the above mentioned panel of wheat (Fig. 5H, Additional file 1: Table S23), as well as in another distinct wheat population (Fig. 5I, Additional file 1: Table S23) and a recombinant inbred line (RIL) population derived from the parents Chinese spring (CS, AA) and Yangmai16 (YM16, GG) (Fig. 5J, Additional file 1: Table S23). On average, the haplotype containing GG alleles exhibited markedly lower seed germination rates and larger seed sizes compared to the haplotype with AA alleles across all three populations (Fig. 5H–J).

In addition, we evaluated the epistatic effects of pyramiding the three synergistic regions (*Qgd-gs.4A.1*, *Qgd-gs.5D.2*, and *Qgd-gs.7A.1*). When all three favorable haplotypes were combined, the beneficial effects significantly increased, yielding improvements of up to 33% for seed dormancy and 12% for seed size (Additional file 2: Fig. S15C).

### Pleiotropic roles of key genes in modulating seed dormancy and seed size

**Fig. 6:**
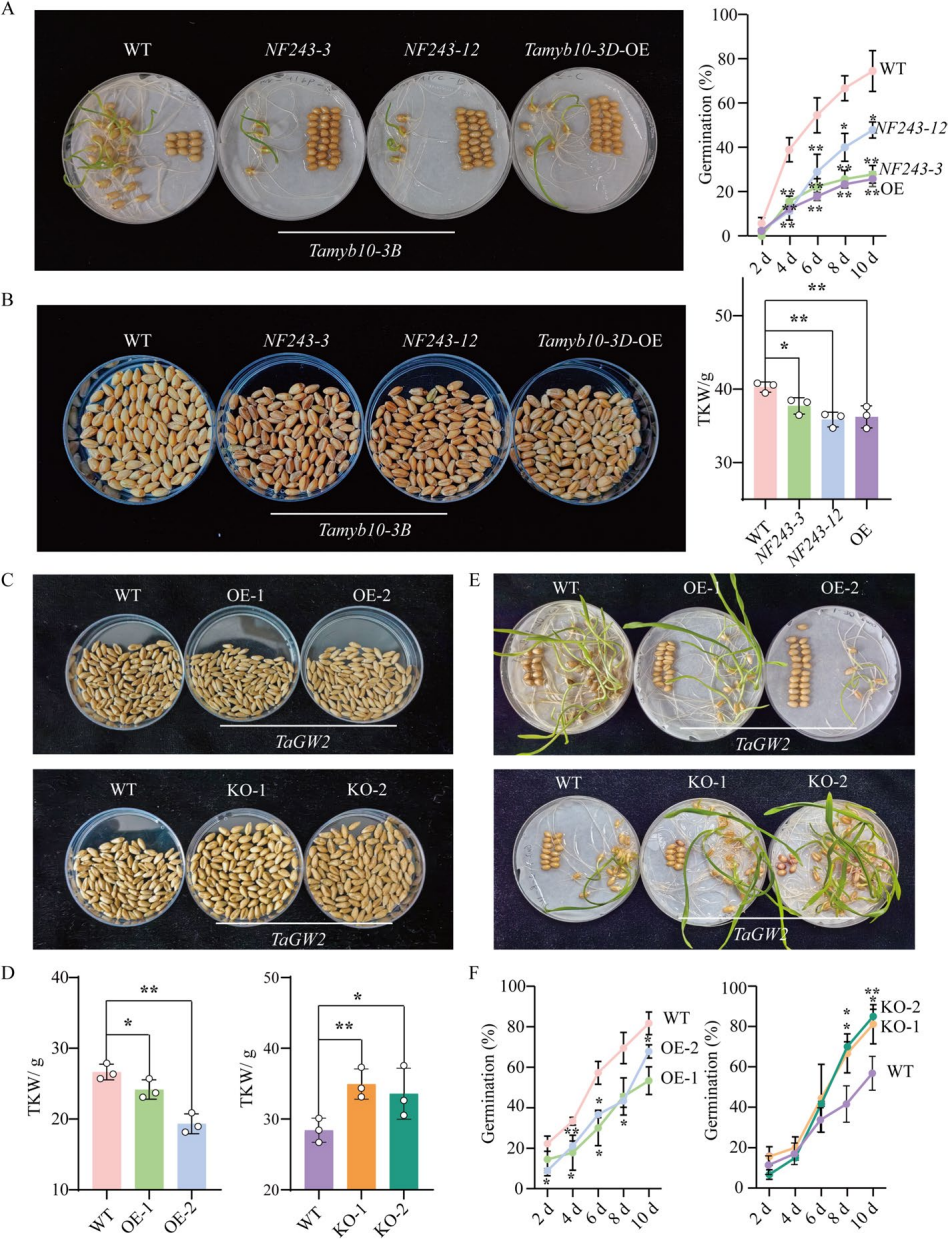
*Tamyb10* and *TaGW2* affect both seed dormancy and seed size in wheat. Germination (**A**) and weight (**B**) phenotypes of *Tamyb10-3D*-overexpressed plants (OE) and *Tamyb10-3B*-edited plants (*NF243-3*, *NF243-12*). Photo in **A** shows the seed germination phenotype of each line. Photo in **B** shows the area of 100 seeds for each line. Values are means ± SE, *n* = 3 biological replicates. Weight (**C**–**D**) and germination (**E**–**F**) phenotypes of *TaGW2-6A*-overexpressed plants (OE-1, OE-2) and *TaGW2-6A* -edited plants (KO-1, KO-2), respectively. Photo in **C** shows the area of 100 seeds for each line. Photo in **E** shows seed germination phenotype of each line. Values are means ± SE, *n* = 3 biological replicates. Significant differences were determined using the Student’s *t*-test. *, 0.01 < *P* < 0.05; **, *P* < 0.01

As shown in Additional file 2: Fig. S13, *Tamyb10-3A* was located in an antagonistic region. To further explore whether *Tamyb10* exhibits pleiotropy, *Tamyb10-3D*-overexpressed plants (OE) [10] and *Tamyb10-3B*-edited plants (*NF243-3*, *NF243-12*) [11] were analyzed. As expected, the germination of both the OE line and gene-edited lines was delayed compared to WT (Fig. 6A), and seed color of the OE line and gene-edited lines changed from white to red (Fig. 6B). Notably, the TKW of OE line and gene-edited lines were significantly reduced compared to the WT (Fig. 6B). Furthermore, based on seed coloration, 545 worldwide wheat accessions were divided into white seeds and red seeds. Phenotypic association analysis revealed that white wheat had weak dormancy and large seed size, while red wheat exhibited strong dormancy and small seed size (Additional file 2: Fig. S16). Similarly, due to the antagonistic effects observed between the haplotypes of *TaGW2*, we selected the overexpressing (OE-1, OE-2) and gene-edited plants (KO-1, KO-2) of *TaGW2* [16] for further investigation into the relationship between seed size and dormancy. As anticipated, overexpression of *TaGW2* in wheat resulted in significantly reduced TKW and increased seed dormancy, while knockout of *TaGW2* led to a marked increase in TKW and decreased seed dormancy (Fig. 6C–F). Furthermore, we evaluated the epistatic effects of pyramiding the three genes *Tamyb10-3B*, *Tamyb10-3D*, and *TaGW2-6A* (Additional file 2: Fig. S17). Results showed that a significant improvement in both seed dormancy and seed size was observed only when the favorable haplotype of *Tamyb10-3D* was combined simultaneously with the intermediate haplotype of *TaGW2-6A* and *Tamyb10-3B*. This improvement reached 12% compared to accessions lacking favorable haplotypes or possessing only a single favorable haplotype.

### Haplotype distribution to balance the antagonistic effects between seed dormancy and seed size under different climate condition

**Fig. 7:**
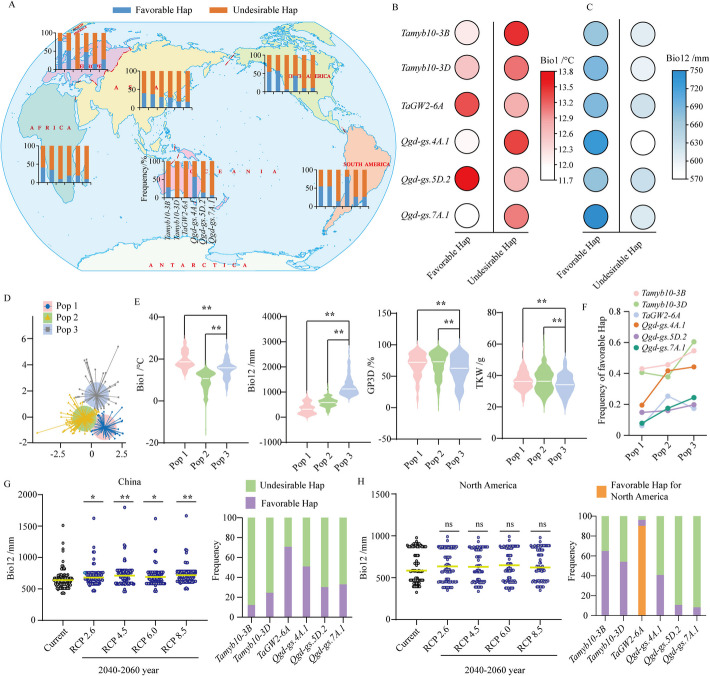
Haplotype distribution and frequency are associated with climate.** A** Geographic distribution of favorable and undesirable haplotypes for three pleiotropic genes (*Tamyb10-3B/D* and *TaGW2-6A*) and three synergistic loci (*Qgd-gs.4A.1*, *Qgd-gs.5D.2*, and *Qgd-gs.7A.1*). **B**–**C** Comparison of favorable and undesirable haplotype frequencies for pleiotropic genes and synergistic loci in relation to Bio1 (annual mean temperature, **B**) and Bio12 (annual precipitation, **C**). **D** Based on Bio1 and Bio12, the 545 wheat accessions were clustered into three populations (Pops). **E** Comparison of the three Pops for Bio1, Bio12, GP3D, and TKW. **F** Frequencies of favorable haplotypes for pleiotropic genes and synergistic loci across three populations.** G**–**H** Under future climate scenarios (RCP 2.6, RCP 4.5, RCP 6.0, and RCP 8.5), precipitation (left panel) and haplotype frequencies for pleiotropic genes and synergistic loci (right panel) in China (**G**) and North America (**H**)*.* Significant differences were determined using the Student’s *t*-test. *, 0.01 < *P* < 0.05; **, *P* < 0.01

Wheat has successfully adapted to a wide range of climates, where environmental factors play a significant role in genetic selection and contribute to genetic variation. As shown in Fig. 7A, the frequency of favorable haplotype for three pleiotropic genes (*Tamyb10-3B/D*, *TaGW2-6A*) and three synergistic regions (*Qgd-gs.4A.1*, *Qgd-gs.5D.2*, and *Qgd-gs.7A.1*) varies across different regions of the world. Wheat yields have declined in response to the combined effects of rising temperature and increasing precipitation variability since the early 1980s [119]. Through analyzing the haplotypes of these pleiotropic genes and synergistic regions in relation to the climatic factors (Bio1, annual mean temperature, and Bio12, annual precipitation) of the original place, it was found that the *Tamyb10-3B/D*, *Qgd-gs.4A.1*, and *Qgd-gs.7A.1* were significantly associated with Bio12 (Fig. 7B–C, Additional file 1: Table S24, Additional file 2: Fig. S18A). Moreover, based on the Bio1 and Bio12 of original locations for 545 wheat accessions, these accessions could be clustered into three populations (Pop; Fig. 7D). Among them, Pop 3 had the highest annual precipitation but exhibited lower GP3D and TKW (Fig. 7E), possibly due to artificial selection for local climatic conditions. As expected, the frequency of favorable haplotype for *Tamyb10-3B/D*, *Qgd-gs.4A.1*, *Qgd-gs.5D.2*, and *Qgd-gs.7A.1* increased with higher Bio12, while the frequency for *TaGW2-6A* decreased (Fig. 7F).

More importantly, our analysis revealed that wheat ecological zones in China are projected to experience heavy precipitation (Fig. 7G, Additional file 1: Table S25, Additional file 2: Fig. S18B) under future different climate scenarios (RCP 2.6, 4.5, 6.0, and 8.5). However, the favorable haplotype frequency of *Tamyb10-3B/D* was found to be significantly lower than that of the undesirable haplotypes (Fig. 7G). In contrast, the favorable haplotype frequency of *Tamyb10-3B/D* is significantly higher than that of the undesirable haplotypes in North America (Fig. 7H), even no significant difference was found in the future precipitation of wheat zones compared to the current climate scenarios (Fig. 7H, Additional file 2: Fig. S18C). Contrary to Chinese cultivar wheat, the favorable and predominant haplotype of *TaGW2-6A* in North American wheat cultivars is Hap 2 with lower GP and higher TKW (Fig. 7H, Additional file 2: Fig. S10C). In addition, it is worth noting that the frequencies of three synergistic loci are from 30 to 51% in China wheat zones, but only from 8 to 41% in North America wheat zones, indicating a significantly lower frequency in the latter (Fig. 7G–H).

## Discussion

Simultaneous optimization of key agronomic traits through traditional breeding has significantly enhanced crop productivity in the past few decades [120]. It has been reported that modern wheat breeding synergistically improved both above- and below-ground traits by selecting haplotype with synergistic effects, ultimately leading to higher yields [121]. Seed dormancy and seed size are two important agronomic traits, which have great effects on crop yield [1, 2]. Loss of seed dormancy can cause pre-harvest sprouting (PHS) and non-uniform seedling establishment. Seed size is one of the important factors that determine the yield of cereal crops. However, the genetic basis of antagonistic effects and simultaneous optimization in domesticated crops for these two traits is still an enigma.

### Negative genetic relationship between seed dormancy and seed size

Seeds represent a critical stage in the life history of plants, and their dormancy and size are two key attributes with profound implications for plant survival and fitness [122]. Larger seeds typically give rise to better-nourished and more robust seedlings, which tend to perform better under adverse conditions compared to those from small seeds [123]. Given that large seeds are well provisioned, they may not rely on dormancy to evade unfavorable conditions. Instead, they could benefit from early germination post-maturity, allowing them to maximize growth before the onset of unfavorable conditions [124]. Early germination of large-seeded seedlings can facilitate the development of deeper root systems, enhancing their ability to access water and nutrients, and strengthening defenses against herbivores and climatic stress [124]. However, this strategy is not without constraints. Larger seeds are more attractive to predators, increasing the risk of predation [125]. In contrast, dormancy can be an adaptive strategy in unpredictable environments, protecting seeds until optimal germination conditions occur. Thus, while large seeds may forgo dormancy to capitalize on early growth opportunities, this comes at the cost of potential resource limitations and higher predation risks, indicating a clear antagonistic effects between seed size and dormancy [122].

However, the genetic basis underlying this selection remains unclear in crop. Herein, benefiting from large-scale sampling and a whole-genome resequencing, our work demonstrated that a significant phenotypic change characterized by decreased seed dormancy and increased seed size was observed during the transition from wheat landraces to cultivars. MTA SNPs and lead SNPs associated with seed dormancy exhibited decreased FAF, while MTA SNPs and lead SNPs linked to seed size showed increased FAF in cultivars compared to landraces. The average values of seed size tended to decrease with the increasing number of seed dormancy favorable alleles. Conversely, the average values of seed germination tended to increase with the increasing number of seed size favorable allele. Moreover, seed dormancy traits were significantly correlated with seed size traits both phenotypically and genetically. These results indicated that small and large seeds in common wheat exhibit a significantly negative correlation in dormancy levels, consistent with the findings from wild emmer to domesticated emmer wheat [126]. Favorable alleles of seed dormancy and seed size were oppositely selected during wheat breeding, resulting in a negative genetic relationship between the two traits. Our findings suggest that the combination of large seed/weak seed dormancy has been artificially heavily selected during wheat breading improvement, potentially increasing PHS risk when harvest season coincides with rainy weather.

### Gene pleiotropy and genetic linkage restrict simultaneous optimization for seed dormancy and seed size

Pleiotropy occurs when a gene influences multiple traits, contributing to synergistic or antagonistic genetic relationships among traits. It was reported that seed dormancy was regulated by many flowering-time genes such as *AtFLC* and *AtFCA* [127], indicating that dormancy can be regulated by pleiotropic genes to adapt to environmental conditions. Recently, a major seed dormancy QTL *SD6* was identified to show antagonistic pleiotropy between seed dormancy and seed size. Knockout of *SD6* displayed significantly increased resistance to PHS but reduced seed size in rice cultivars Zhonghua11 and Tianlong619 [1]. *NF-YB1*, a rice endosperm-specific core transcription factor, also played an antagonistic role in regulating seed dormancy and seed size traits [128]. Herein, we provide robust evidences that gene pleiotropy plays a crucial role in the negative genetic relationship between seed dormancy and seed size. First, haplotype analysis revealed that 10 out of 18 known seed dormancy-related genes and 11 out of 18 known seed size-related genes exhibit antagonistic effects on seed dormancy and seed size. These genes include *Tamyb10*, *TaMFT*, *TaMKK3*, *TaSD6* and *TaGW2*, *TaPGS1*, *TaARF25*, and *TaSPL17*. Second, gene-edited and/or overexpressed plants of major gene *Tamyb10* and *TaGW2* confirmed their pleiotropy with antagonistic effects on seed dormancy and seed size. Specifically, *Tamyb10*-overexpressed and *Tamyb10-*edited plants enhance seed dormancy and seed color [10, 11], but distinctly reduces seed size. *TaGW2* negatively impacts seed size [16, 17], but positively regulates seed dormancy. Due to gene pleiotropy, allelic substitutions at one locus can affect multiple traits, making them resistant to change even under strong selection [129]. Undoubtedly, these pleiotropic genes have exhibited a preference for selecting haplotypes with higher seed size during wheat breeding, leading to the negative genetic relationship between seed dormancy and seed size. In this context, it may be advantageous to explore intermediate haplotypes of seed dormancy- or seed size-related genes for wheat breeding or to focus selection efforts on loci that are not regulated by pleiotropy. Interestingly, haplotype analysis revealed that favorable alleles of two genes, *TaDA1* and *TaAFP*, exhibit synergistic effects, conferring deeper seed dormancy and larger seed size. This finding could be very helpful for future wheat breeding improvement.

Recent studies have demonstrated that QTLs affecting fitness traits in crops are frequently clustered within the genome, indicating that tight genetic linkage may exert complex effects on multiple components of fitness [130, 131]. In emmer wheat, *QGD-4BL* was significantly associated with the natural variation of seed size and seed dormancy, which could be controlled by a single gene with pleiotropic effects or tightly linked genes [132]. It was found that numerous seed dormancy-related regions/genes overlap with known seed size-related QTLs/genes in common wheat. *TaAFP-2B* overlaps with the loci for seed number per spike (*QGns.saas-2B*) and seed yield per plot (*Qgy.tamu.2B.1.1*) [46]. *TaMFT-3A* overlaps with loci for TKW such as *QTgw.cau-3A.1* [82], *QTKW.ndsu.3A.1* [84], *QTkw-3A.1* [85], and *qTKW-3A.1* [86]. In our study, 66 regions for seed dormancy were detected with overlapping confidence intervals with seed size, and among them, 21 regions exhibited significant antagonistic effects on seed dormancy and seed size. The strong selection during wheat breeding improvement has led to the fixation of favorable alleles for seed size at these antagonistic linkage loci, thereby reducing the heritability of seed dormancy. Furthermore, we found that a significantly higher proportion of MTA SNPs (18.59%–28.60%) and lead SNPs (13.61%–32.39%) harbored antagonistic alleles than synergistic ones. This prevalence of antagonistic loci, which collectively explained up to 26.56% (GP) and 47.21% (TKW) of the phenotypic variation, reveals a strong negative genetic relationship. Consequently, optimizing seed dormancy through the accumulation of favorable, non-antagonistic haplotypes appears achievable, but breaking this negative correlation to improve seed size will be considerably more difficult. Overall, these results strongly support the notion that synchronous antagonistic selections at loci or linkage loci related to seed dormancy and seed size have significantly shaped the agronomic traits of modern wheat during breeding improvement. Large seed size is the main target of breeding efforts [133], which compromises the seed dormancy trait. There were three overlapping loci with dramatically synergistic effects on seed dormancy and seed size (higher TKW and lower GP). These three loci represent valuable genetic resources for balancing and enhancing seed size and dormancy in future wheat breeding improvement. Based on the haplotype analysis and molecular marker, we identified a candidate gene *RNA-binding protein* (*TaRBP-4A*) for synergistic locus *Qgd-gs.4A.1*, which may improve both seed dormancy and seed size traits. In plants, RBPs play crucial roles in various biological processes, including seed germination, root growth, stem cell maintenance, flowering, and seed development, through their influence on RNA metabolism [134].

### Antagonistic selection between seed dormancy and size responding to climate adaption and change

Local adaptation is a critical factor in responding to changing environments, as it illustrates how environmental changes can drive phenotypic and genetic differentiation through selection [135]. Precipitation has a complex effect on PHS characteristics in cereal crops. In East Asia, the rainy season often coincides with the cereal harvest season, leading to serious PHS disasters [1]. Moreover, seeds exhibiting PHS show heightened activity of oxidoreductases and hydrolases, leading to depletion of nutrients (starch, protein, and oil) and increased susceptibility to spoilage [136]. A recent study demonstrated that the minor allele frequencies of SNPs in dormancy-related genes increased along with the spread of wheat, which is consistent with trends in rain and humidity [137]. Similarly, in rice, the distribution frequency of the *Sdr4-n* allele in different locations in Asia is significantly associated with local annual precipitation [138]. Consistent with previous findings, our results indicated that favorable variants of *Tamyb10* and three synergistic loci (*Qgd-gs.4A.1*, *Qgd-gs.5D.2*, and *Qgd-gs.7A.1*) have significantly expanded and spread to areas with heavy rainfall under the selective pressure of precipitation, likely due to the preference for deeply dormant seeds in humid regions. Notably, favorable haplotypes of *Tamyb10* are frequently utilized to maintain appropriate seed dormancy in North America, but not in China. However, the three synergistic loci are more commonly utilized in China, though their FAF being less than 51%, suggesting substantial potential for these loci to improve seed dormancy and seed size in common wheat both in North America and China. Additionally, our work indicates that the favorable and predominant haplotype of *TaGW2-6A* in North America wheat cultivars is Hap 2 (with lower GP and higher TKW) rather than Hap 1, consistent with the difference in favorable and predominant haplotype of *TaGW2-6A* between China and America [139]. On the one hand, this difference might be attributed to variations in flowering and maturity time between the two haplotypes [139]. On the other hand, it could result from differences in dormancy levels between the two haplotypes. These results suggest that breeding selection in the two regions has been achieved by targeting the same genes but utilizing different types of variants. In the future, wheat ecological zones in China are expected to experience heavier precipitation due to global warming and extreme rainfall events, leading to more frequent PHS. Therefore, developing wheat varieties with synergistic alleles that balance seed dormancy and seed size will be an effective strategy for adapting to future global climate changes.

## Conclusions

This study constructed a genome-wide map of genetic variations associated with seed dormancy and seed size traits, providing genomic insights into the impacts of antagonistic effects on both seed dormancy and seed size. These insights were derived from large-scale sampling and whole-genome resequencing in common wheat. The identification of genomic regions or genes under balancing selection between seed dormancy and seed size traits will facilitate the development of genetic resource toolkits for the bidirectional and reciprocal improvement of wheat in the future.

## Methods

### Planting and phenotyping

These 545 wheat accessions (Additional file 1: Table S1) were planted during the winter growing seasons at an experimental farm in Zhaoxian, Hebei, China (year 2021, 2022, and 2023) and Xiangshan, Beijing, China (year 2023). Each location included two replicates to ensure data repeatability. A randomized complete block design was employed (Additional file 1: Table S26). In each replicate, accessions were randomly arranged in six rows with a row and column spacing of 150× 10 cm, and each row included 15 plants. Standard field management practices (e.g., irrigation, weed management, fertilization) were performed. Plants were irrigated as needed. Approximately 15 to 20 physiologically mature plants, characterized by the loss of green pigmentation in the spike and peduncles, were collected and air-dried for 3 days.

Seed dormancy was evaluated by calculating the germination percentage (GP/%) based on the number of freshly harvested seeds that germinated after 3 days (first count germination, GP3D) and 7 days (final germination, GP7D) of imbibition. Approximately 100 fresh seeds were placed on moist filter papers in petri dishes under 16-h light (23 °C)/8-h dark (16 °C) conditions for germination tests [44]. Germination was defined as the emergence of radicle breaking through the seed coat. The GP values for the 545 wheat accessions in four different environments were measured and named Zhaoxian, 2021 (ZX2021), Zhaoxian, 2022 (ZX2022), Zhaoxian, 2023 (ZX2023), and Beijing, 2023 (BJ2023). For seed size traits, three character traits were quantified, including thousand kernel weight (TKW/g), kernel length (KL/mm), and kernel width (KW/mm). The genetic correlation between seed dormancy and seed size traits was determined using the GREML function of GCTA software (https://yanglab.westlake.edu.cn/software/gcta).

*Tamyb10-3D*-overexpressed plants (OE) were developed by Lang et al. [10]. *Tamyb10-3B*-edited plants (*NF243-3*, *NF243-12*) were provided by Zhu et al. [11]. *TaGW2*-overexpressed plants (OE-1, OE-2) and gene-edited plants (KO-1, KO-2) were generated by Liu et al. [16]. Detailed genetic transformation methods for the *Tamyb10* and *TaGW2* genes are described in previous reports [10, 11, 16]. WT (cv. Fielder) and transgenic or gene-edited plants were grown in the greenhouse under 14-h light (23 °C)/10-h dark (18 °C) conditions. For the determination of TKW and germination rate, at least 15 overexpressed plants and gene-edited plants were randomly selected.

### BLUE

To obtain the BLUE values and eliminate environmental effects from the analysis, BLUE values of GP, TKW, KL, and KW across all environments were calculated using the lme4 package in the R v4.1.2 (http://www.r-project.org/) with the linear mixed linear model:

$$Yijkl =\mu + Gi + Lj + Yk + (GL)ij + (GY)ik + (LY)jk + (GLY)ijk +\epsilon ijkl$$

where *Y*_*ijkl*_ is the trait value of the *i*-th genotype at the *j*-th location, in the *k*-th year, and for the *l*-th replicate. *μ* is the mean. *G*_*i*_ is the effect of the *i*-th genotype. *L*_*j*_ is the effect of the *j*-th location. *Y*_*k*_ is the effect of the *k*-th year. (*GL*)_*ij*_ is the interaction effect between the genotype and the location. (*GY*)_*ik*_ is the interaction effect between the genotype and the year. (*LY*)_*jk*_ is the interaction effect between the location and the year. (*GLY*)_*ijk*_ is the interaction effect among the genotype, the location, and the year. *ϵ*_*ijkl*_ is the random error term, accounting for the influence of other random factors on the observed value not explained by the above factors.

### Broad sense heritability

Phenotypic data from all environments were analyzed by ANOVA in R. The broad sense heritability (*H*^2^) of GP3D, GP7D, TKW, KL, and KW was calculated across environments based on variance components according to the following formula:

$$H^2=(\sigma^2 G)/(\sigma^2 G+(\sigma^2 GL)/L+(\sigma^2 GY)/Y+(\sigma^2 E)/YRL)$$

where *σ*^*2*^_*G*_ is the genotypic variance, *σ*^*2*^_*GL*_ is the genotype by the location effect, *σ*^*2*^_*GY*_ is the genotype by the year effect, *σ*^2^_*E*_ is the residual error, *L* means location (*L* = 2), *Y* means year (*Y* = 3), and *R* means the number of replications (*R* = 2).

### Genotype calling and SNP identification

SNP discovery and genotyping were conducted following the established workflow for the construction of high-density genetic variation map [140–142]. The high-density genetic variation map dataset of 545 wheat accessions was generated following the stringent sample quality control by site and taxon. In total, 63,379,140 segregating SNPs (minimum allele frequency [MAF] > 0.05; missing rate < 20%; missing genotype rate < 10%) were utilized for GWAS.

### GWAS

GWAS were conducted to investigate the genetic basis of seed dormancy and seed size using 545 worldwide wheat accessions, 265 landrace wheat accessions, and 215 cultivar wheat accessions, respectively. Based on 63,379,140 SNPs, GWAS was preformed using a mixed linear model (MLM) with efficient mixed model association expedited (EMMAX) software [143]. The kinship matrix and the first PCA were utilized as the random effect and fixed effect covariates, respectively. Bonferroni correction of 1/*n* was applied, where *n* represents the number of makers. To balance false positives and false negatives, a moderate number of independent markers were conservatively chosen using PLINK (v1.9.0) software [144]. Finally, a significant threshold of − log_10_ (*P-*value) = 5.0 was set for calling significant associations. Visualization of GWAS results was achieved through Manhattan plots generated using the CMplot package in R v4.1.2 (https://github.com/YinLiLin/R-CMplot). The number of significant SNPs within each genomic region was counted. If a genomic region contained more than five significant SNPs, it was defined as an associated genomic region. The SNP with the lowest *P*-value within each associated genomic region was designated as the lead SNP representing the particular region. Phenotypic comparisons between two haplotypes were performed by *t*-tests.

### Selective signals and selective sweep scanning for wheat breeding improvement

To identify selective signals in wheat breeding improvement, π and *F*st levels between landraces, cultivars, and other wheat accessions were calculated using 200-kb sliding windows with a step of 100 kb by VCFtools (v0.1.14) [145]. The XP-CLR score was used to scan for selective sweep regions during modern wheat improvement, with the parameters “–ld 0.95 –maxsnps 1000 –size 50,000 –step 20,000” [146]. Candidate regions undergoing selective sweeps during breeding improvement were identified based on the top 5% of *F*st, π ratio (π_Landraces_/π_Cultivars_), or XP-CLR scores.

### Phylogenetic tree and population structure

Consider the large LD distance and high-density SNP distribution, we obtained a random subset of relatively independent SNPs using the PLINK (v1.9.0) with the parameters “–indep-pairwise 1000 10 0.4.” The population genetic structure was conducted using the pruned subset SNPs with the Admixture program (https://dalexander.github.io/admixture/). The number of assumed genetic clusters *K* ranged from 1 to 13, with 10,000 iterations for each run. A total of 13 independent runs of Admixture with different random seeds were performed at each *K* value. The phylogenetic tree was constructed based on neighbor-joining method using the FastTree (http://www.microbesonline.org/fasttree/) and visualized using ITOL (https://itol.embl.de/). PCA was also performed to evaluate genetic structure using the PLINK (v1.9.0) software. PCA was carried out to remove linked SNPs based on LD blocks, resulting in 3,702,062 SNPs for population structure analysis.

### LD analysis

To estimate and analyze the LD decay patterns for all samples and different population, 1% of all SNPs were randomly selected using the parameter “–thin 0.01” in PLINK (v1.9.0). Subsequently, the squared correlation coefficient (*r*^2^) between pairwise SNPs was calculated using PopLDdecay (https://github.com/BGI-shenzhen/PopLDdecay) with the parameter “-MaxDist 1000.”

### Favorable alleles for seed dormancy and seed size traits

Based on the GWAS results, we analyzed the FAF for MTA SNPs (− log_10_ [*P*-value] > 5) and lead SNPs associated with seed dormancy and/or seed size. SNPs associated with reduced GP or increased TKW, KL, and KW were identified as favorable allele, respectively. The changes in FAF between landraces and cultivars were further analyzed. The ratio of increase (for TKW) or decrease (for GP) in the median value was calculated to quantify the genetic effect between alleles.

### Haplotype construction for loci

Haplotype analyses were carried out based on the causal polymorphisms of the corresponding candidate genes using the geneHapR package [147] in the R v4.1.2. Haplotypes with fewer than ten accessions were excluded. Following Liu et al. [148], the SNP-based haplotype construction for overlapping regions associated with seed dormancy and seed size trait was evaluated using the LDheatmap (https://github.com/SFUStatgen/LDheatmap) and Pheatmap (https://github.com/raivokolde/pheatmap) software package in R. TagSNPs were extracted using PLINK (v1.9.0).

### RNA-seq data, climate data, and primers

The RNA-seq data of wheat varieties with different dormancy levels (strong dormancy, Baipimai, Shengsimai; weak dormancy, Zhoumai 18), at different germination time points and developmental stages of wheat tissues, were downloaded from the PlantExp database (https://biotec.njau.edu.cn/plantExp). The expression data for embryo and endosperm in developing seeds were downloaded from WheatOmics 1.0 (http://202.194.139.32/expression/wheat.html). RNA-Seq analysis was performed using dormant or dormancy-released seeds of Darius, a variety with strong seed dormancy (Additional file 2: Fig. S19, Additional file 1: Table S27).

Climate-related variables’ data information was downloaded from WorldClim (https://www.worldclim.org/), providing monthly climate precipitation and temperature data at 2.5-min resolution for the period 1970–2000 and future climate projections. Geographic data analysis was performed using the EXTRACT function of R package RASTER v.3.3.13 (https://cran.r-project.org/web/packages/raster). To assess whether the pleiotropic genes and genome regions are correlated with the precipitation or the annual average temperature, Student’s *t*-tests were conducted between the favorable haplotypes and the undesirable haplotypes for *Tamyb10-3B/D*, *TaGW2*, *Qgd-gs.4A.1*, *Qgd-gs.5D.2*, and *Qgd-gs.7A.1*. The primer sequences for gene markers are listed in Additional file 1: Table S28.

## Supplementary Information

Additional file 1: Table S1–S29. This file contains all supplementary tables. Table S1 Summary of the 545 worldwide wheat accessions. Table S2 Seed dormancy and seed size traits of the 545 wheat accessions. Table S3 Statistical analysis of seed dormancy and seed size of 545 wheat accessions across environments. Table S4 Detailed information of all significantly associated SNPs for the GP3D traits (−log_10_ [*P*-value] >5). Table S5 Detailed information of all significantly associated SNPs for the GP7D traits (−log_10_ [*P*-value] >5). Table S6 Detailed information of all significantly associated SNPs for the TKW traits (−log_10_ [*P*-value] >5). Table S7 Detailed information of all significantly associated SNPs for the KL traits (−log_10_ [*P*-value] >5). Table S8 Detailed information of all significantly associated SNPs for the KW traits (−log_10_ [*P*-value] >5). Table S9 Summary of associated genomic regions information for seed dormancy and seed size traits by GWAS. Table S10 Haplotype analysis of candidate genes *TaPP2C-4A*, *TaGATA54*, *TaPdxB-4A* and *TaRBP-4A* in each of wheat accessions. Table S11 Selection signatures and sweeps detected between landraces and cultivars. Table S12 Genes in selection signatures and sweeps detected between landraces and cultivars. Table S13 Percentage of changed FAF for seed dormancy and seed size between cultivars and landraces. Table S14 The number of favorable alleles for seed dormancy and seed size in each of wheat accessions. Table S15 The associated genomic regions of seed dormancy or seed size overlapped with known loci of seed size or seed dormancy. Table S16 The number of favorable alleles of known seed dormancy genes for each of wheat accessions. Table S17 The number of favorable alleles of known seed size genes for each of wheat accessions. Table S18 Frequency of synergistic alleles, antagonistic alleles and independent alleles in wheat. Table S19 Phenotypic variation explained by synergistic alleles, antagonistic alleles and independent alleles for MTA SNPs in wheat. Table S20 Haplotype analysis of 66 overlapping regions for the result of seed dormancy and seed size by GWAS. Table S21 Bidirectional significance analysis of 66 overlapping regions for seed dormancy and seed size traits. Table S22 Detail information of 66 overlapping regions. Table S23 The SNP allele of different wheat populations base on CAPS marker for *TaRBP-4A*. Table S24 The climate data of 545 wheat accessions in this study. Table S25 The future Bio12 data of cultivar wheat accessions from China and North America. Table S26 The experimental design of growing wheats. Table S27 Differentially expressed genes between dormant seeds and dormancy-released seeds at different imbibition stage in wheat Darius. Table S28 The primer sequence for gene marker in this study. Table S29 Detailed information about acronyms in this study.

Additional file 2: Fig. S1–S19. This file contains all supplementary figures. Fig. S1 Density distribution of SNPs on common wheat chromosomes. Fig. S2 Phenotypic analyses of 545 wheat accessions for seed dormancy and seed size traits. Fig. S3 Genome-wide association analysis of seed dormancy and seed size traits in different environments. Fig. S4 *TaPP2C-4A* and *TaGATA54* were associated with seed dormancy in wheat. Fig. S5 *TaPdxB-4A* was associated with seed size in wheat. Fig. S6 The comparison of two major haplotypes frequency between landraces and cultivars for seed dormancy/size genes targeted by selection. Fig. S7 Breeding selection of favorable alleles and change of FAF. Fig. S8 Relationships between seed dormancy or seed size favorable allele number and phenotypic values. Fig. S9 Phenotypic difference between haplotypes for known seed dormancy genes. Fig. S10 Phenotypic difference between haplotypes for known seed size genes. Fig. S11 The relationships between number of favorable haplotypes and seed dormancy trait or seed size trait. Fig. S12 Genome-wide association analysis of seed dormancy and seed size using landrace (left panel) and cultivar (right panel) wheat accessions. Fig. S13 The overlapping regions between seed dormancy and seed size were identified by GWAS result. Fig. S14 GP3D and TKW difference between favorable and undesirable haplotype for *Qgd-gs.5D.2*and* Qgd-gs.7A.1*. Fig. S15 *TaRBP-4A* was associated with seed dormancy and seed size in wheat and the phenotypic effects of allelic combinations of *Qgd-gs.4A.1*, *Qgd-gs.5D.2* and *Qgd-gs.7A.1* for GP3D and TKW in the 545 wheat accessions collection. Fig. S16 Correlation analysis between seed color and phenotype in wheat. Fig. S17 The phenotypic effects of allelic combinations of *Tamyb10-3B/D* and *TaGW2* for GP3D and TKW in the 545 wheat accessions collection. Fig. S18 Comparison of favorable and undesirable haplotypes of pleiotropic genes and synchronous loci for Bio1 and Bio12 and under future (2080–2100 year) climate scenarios, the precipitation in China and North America will increase. Fig. S19 RNA-seq analysis of dormant seeds and dormancy-released seeds of wheat landrace Darius.

## Data Availability

All scripts used in this work are available at Github under the MIT license (https://github.com/guofeilong1117/wheat-seed-size-and-dormancy.git) [149] together with a Zenodo repository (10.5281/zenodo.16270697) [150]. The RNA-seq data derived from the dormant or dormancy-released seeds of Darius have been deposited in the Genome Sequence Archive (accession number CRA028160) [151]. The VCF data of 545 wheat accession used in this study have been deposited in the Genome Variation Map (accession number GVM001131) [152]. The RNA-seq data for embryo and endosperm at 14 DPA and 25 DPA [153], RNA-seq data for root, stem, leaf, spike and seed of wheat [154] and RNA-seq data for wheat varieties with different dormancy levels [155] were downloaded from published studies, respectively.
