# Supplementary material for: Antagonistic effects of selection on alleles associated with seed size and seed dormancy in wheat
Source: Genome Biol. 2025 Sep 25;26:304. doi: 10.1186/s13059-025-03770-9 (PMC12465254; doi:10.1186/s13059-025-03770-9)
Supplement: Supplementary file 2 — Additional file 2: Fig. S1–S19. This file contains all supplementary figures. Fig. S1 Density distribution of SNPs on common wheat chromosomes. Fig. S2 Phenotypic analyses of 545 wheat accessions for seed dormancy and seed size traits. Fig. S3 Genome-wide association analysis of seed dormancy and seed size traits in different environments. Fig. S4 TaPP2C-4A and TaGATA54 were associated with seed dormancy in wheat. Fig. S5 TaPdxB-4A was associated with seed size in wheat. Fig. S6 The comparison of two major haplotypes frequency between landraces and cultivars for seed dormancy/size genes targeted by selection. Fig. S7 Breeding selection of favorable alleles and change of FAF. Fig. S8 Relationships between seed dormancy or seed size favorable allele number and phenotypic values. Fig. S9 Phenotypic difference between haplotypes for known seed dormancy genes. Fig. S10 Phenotypic difference between haplotypes for known seed size genes. Fig. S11 The relationships between number of favorable haplotypes and seed dormancy trait or seed size trait. Fig. S12 Genome-wide association analysis of seed dormancy and seed size using landrace (left panel) and cultivar (right panel) wheat accessions. Fig. S13 The overlapping regions between seed dormancy and seed size were identified by GWAS result. Fig. S14 GP3D and TKW difference between favorable and undesirable haplotype for Qgd-gs.5D.2and Qgd-gs.7A.1. Fig. S15 TaRBP-4A was associated with seed dormancy and seed size in wheat and the phenotypic effects of allelic combinations of Qgd-gs.4A.1, Qgd-gs.5D.2 and Qgd-gs.7A.1 for GP3D and TKW in the 545 wheat accessions collection. Fig. S16 Correlation analysis between seed color and phenotype in wheat. Fig. S17 The phenotypic effects of allelic combinations of Tamyb10-3B/D and TaGW2 for GP3D and TKW in the 545 wheat accessions collection. Fig. S18 Comparison of favorable and undesirable haplotypes of pleiotropic genes and synchronous loci for Bio1 and Bio12 and under future (2080–2100 y [file 13059_2025_3770_MOESM2_ESM.pdf]

Description of Additional file 2.

Fig. S1 Density distribution of SNPs on common wheat chromosomes.

Fig. S2 Phenotypic analyses of 545 wheat accessions for seed dormancy and seed size traits.

Fig. S3 Genome-wide association analysis of seed dormancy and seed size traits in different environments.

Fig. S4 *TaPP2C-4A* and *TaGATA54* were associated with seed dormancy in wheat.

Fig. S5 *TaPdxB-4A* was associated with seed size in wheat.

Fig. S6 The comparison of two major haplotypes frequency between landraces and cultivars for seed dormancy/size genes targeted by selection.

Fig. S7 Breeding selection of favorable alleles and change of FAF.

Fig. S8 Relationships between seed dormancy or seed size favorable allele number and phenotypic values.

Fig. S9 Phenotypic difference between haplotypes for known seed dormancy genes.

Fig. S10 Phenotypic difference between haplotypes for known seed size genes.

Fig. S11 The relationships between number of favorable haplotypes and seed dormancy trait or seed size trait.

Fig. S12 Genome-wide association analysis of seed dormancy and seed size using landrace (left panel) and cultivar (right panel) wheat accessions.

Fig. S13 The overlapping regions between seed dormancy and seed size were identified by GWAS result.

Fig. S14 GP3D and TKW difference between favorable and undesirable haplotype for *Qgd-gs.5D.2* and *Qgd-gs.7A.1*.

Fig. S15 *TaRBP-4A* was associated with seed dormancy and seed size in wheat and the phenotypic effects of allelic combinations of *Qgd-gs.4A.1*, *Qgd-gs.5D.2 and Qgd-gs.7A.1* for GP3D and TKW in the 545 wheat accessions collection.

Fig. S16 Correlation analysis between seed color and phenotype in wheat.

Fig. S17 The phenotypic effects of allelic combinations of *Tamyb10-3B/D* and *TaGW2* for GP3D (A) and TKW (B) in the 545 wheat accessions collection.

Fig. S18 Comparison of favorable and undesirable haplotypes of pleiotropic genes and synchronous loci for Bio1 and Bio12 (A) and under future (2080-2100 year) climate scenarios, the precipitation in China (B) and North America (C) will increase.

Fig. S19 RNA-seq analysis of dormant seeds and dormancy-released seeds of wheat landrace Darius.

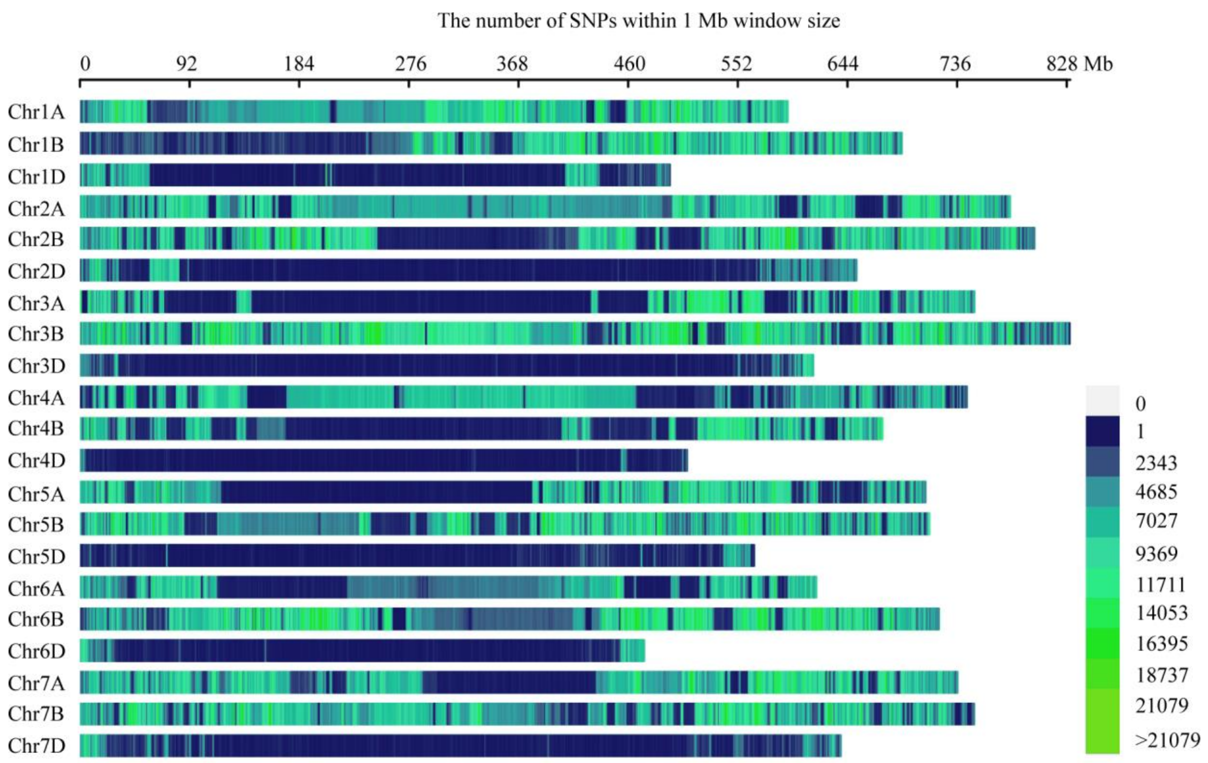

Fig. S1 Density distribution of SNPs on common wheat chromosomes.

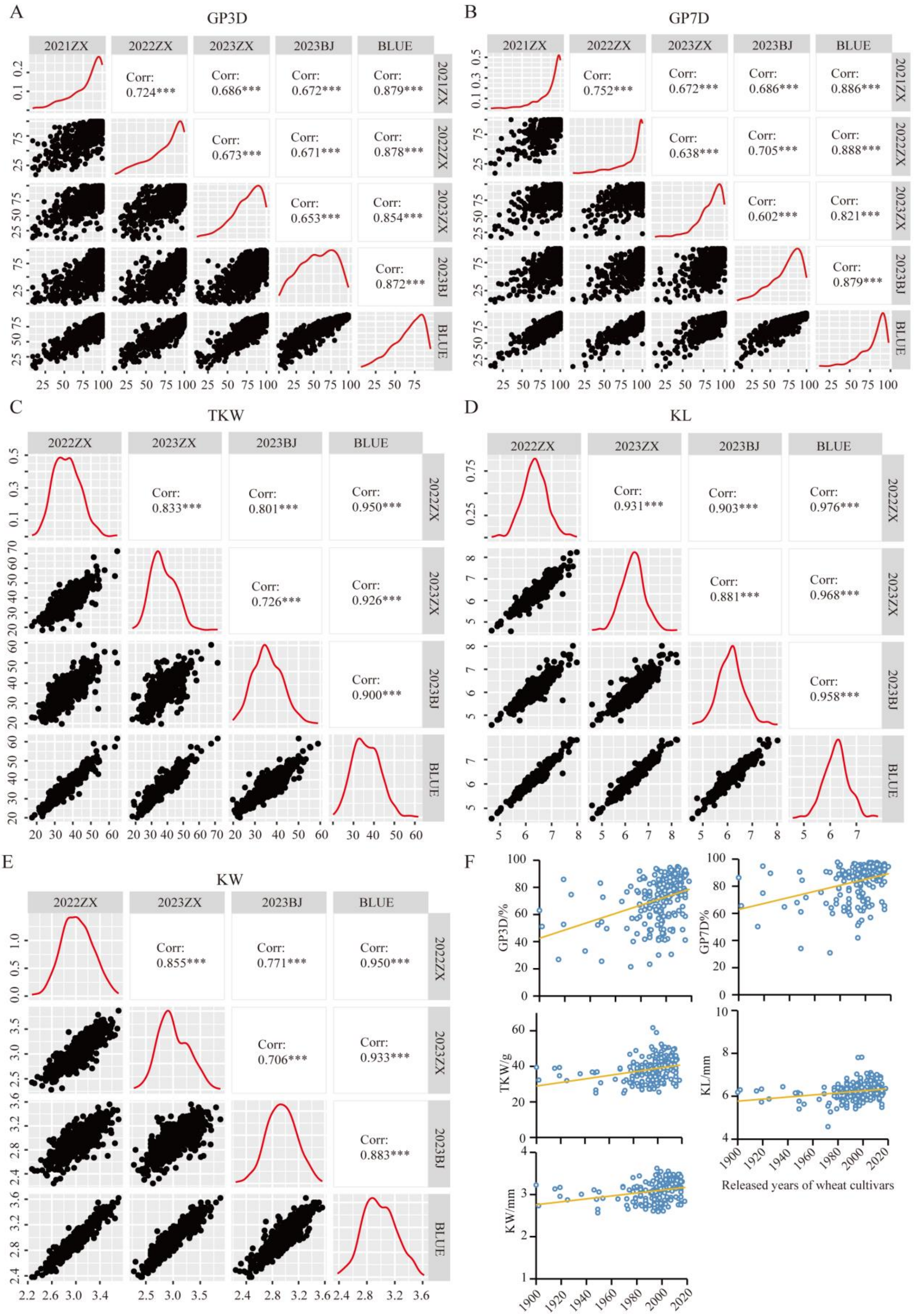

Fig. S2 Phenotypic analyses of 545 wheat accessions for seed dormancy and seed size traits. Pearson's correlation coefficients were calculated using the phenotypic BLUE values of GP3D (A), GP7D (B), TKW (C), KL (D) and KW (E). ZX indicated Zhaoxian, BJ indicated Beijing. (F) The change trend of GP3D, GP7D, TKW, KL and KW phenotypic values of cultivar released in different periods.

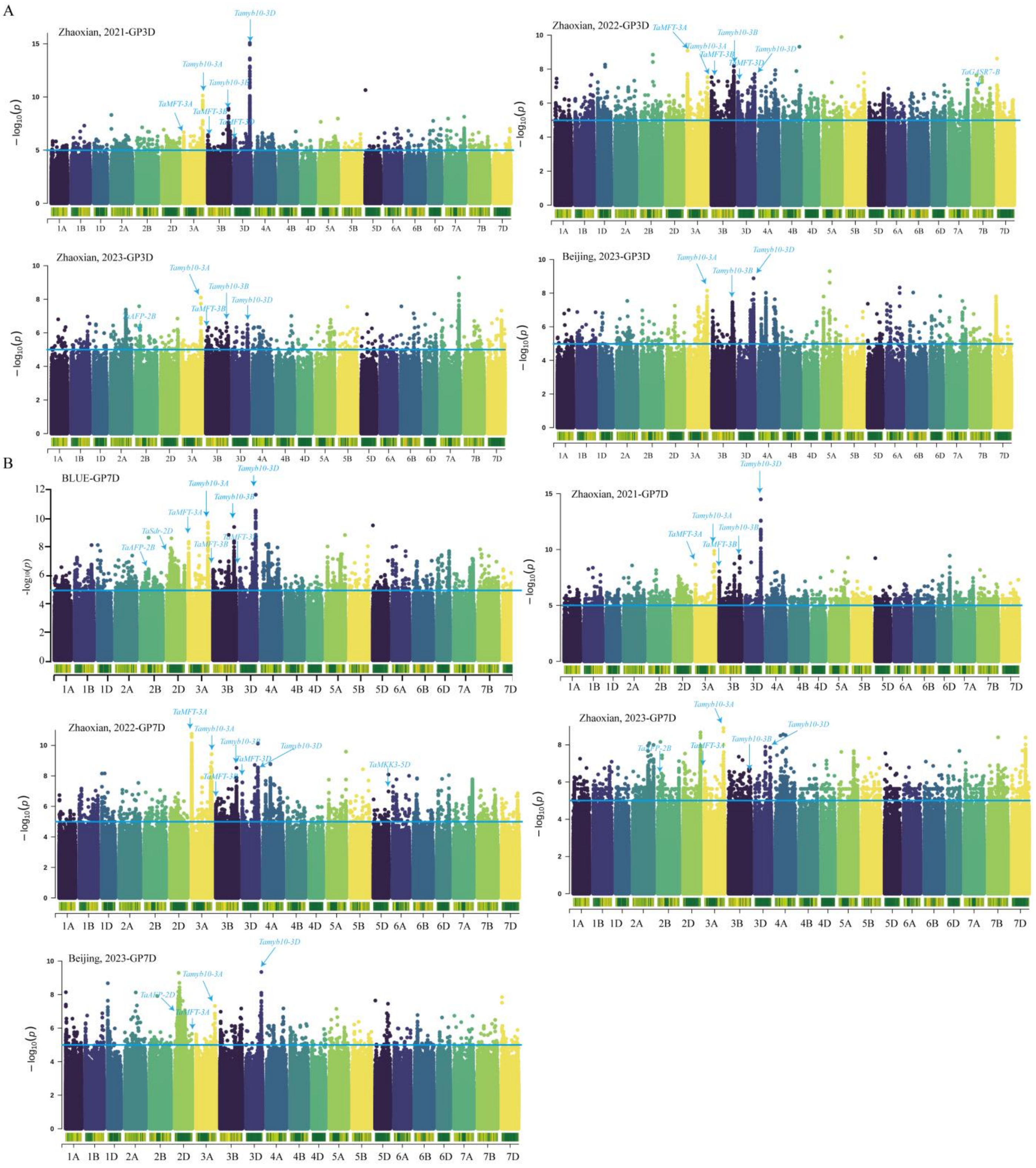

Fig. S3A-B Genome-wide association analysis of seed dormancy and seed size traits in different environments. Manhattan plots showing the SNP marker-trait associations for the GP3D (A) and GP7D (B). Blue arrows represented known seed dormancy genes. Blue line indicated the significance threshold (-log[*P*-value]>5).

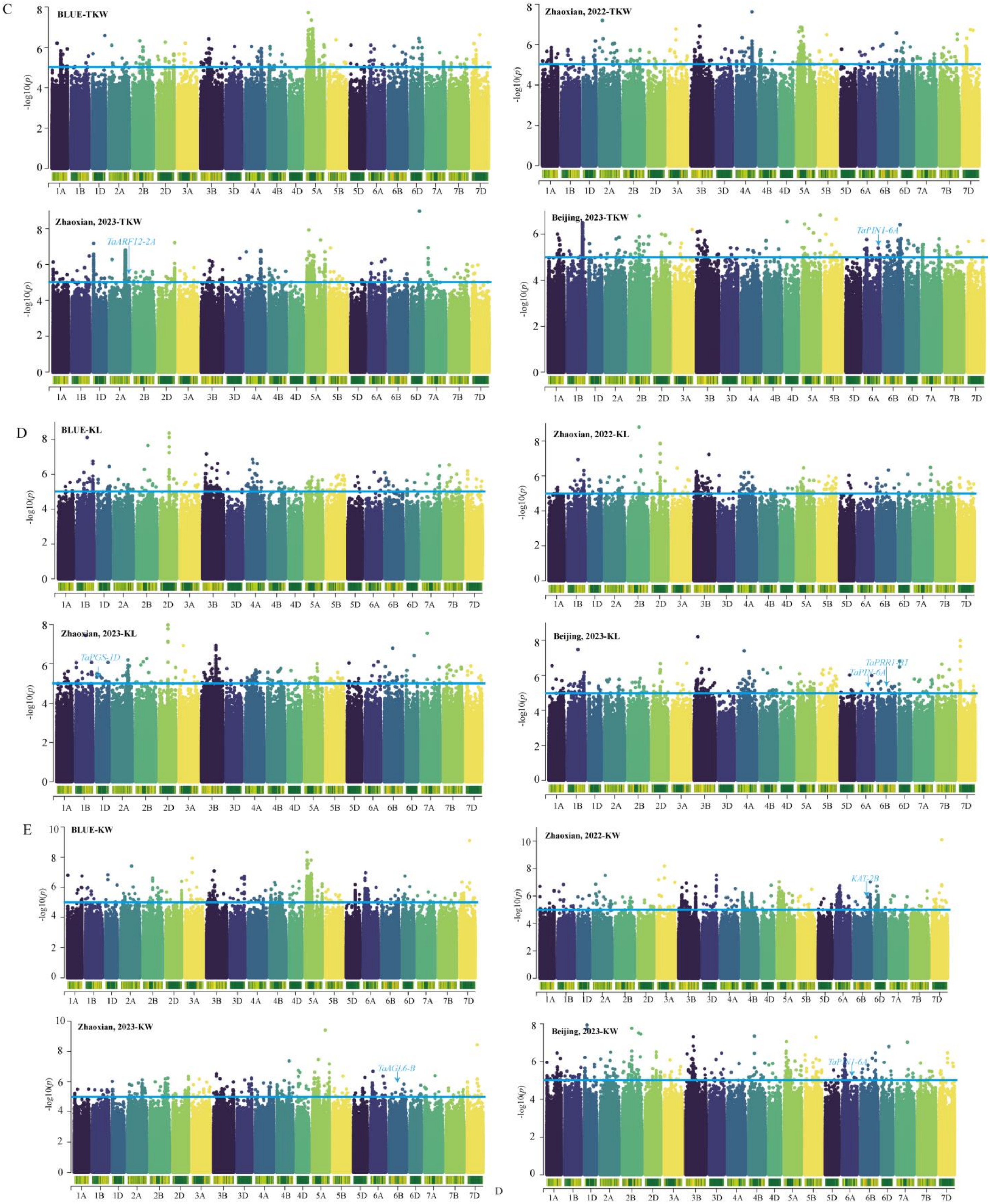

Fig. S3C-E Genome-wide association analysis of seed dormancy and seed size traits in different environments. Manhattan plots showing the SNP marker-trait associations for the TKW (C), KL (D) and KW (E). Blue arrows represented known seed size genes. Blue line indicated the significance threshold (-log[*P*-value]>5).

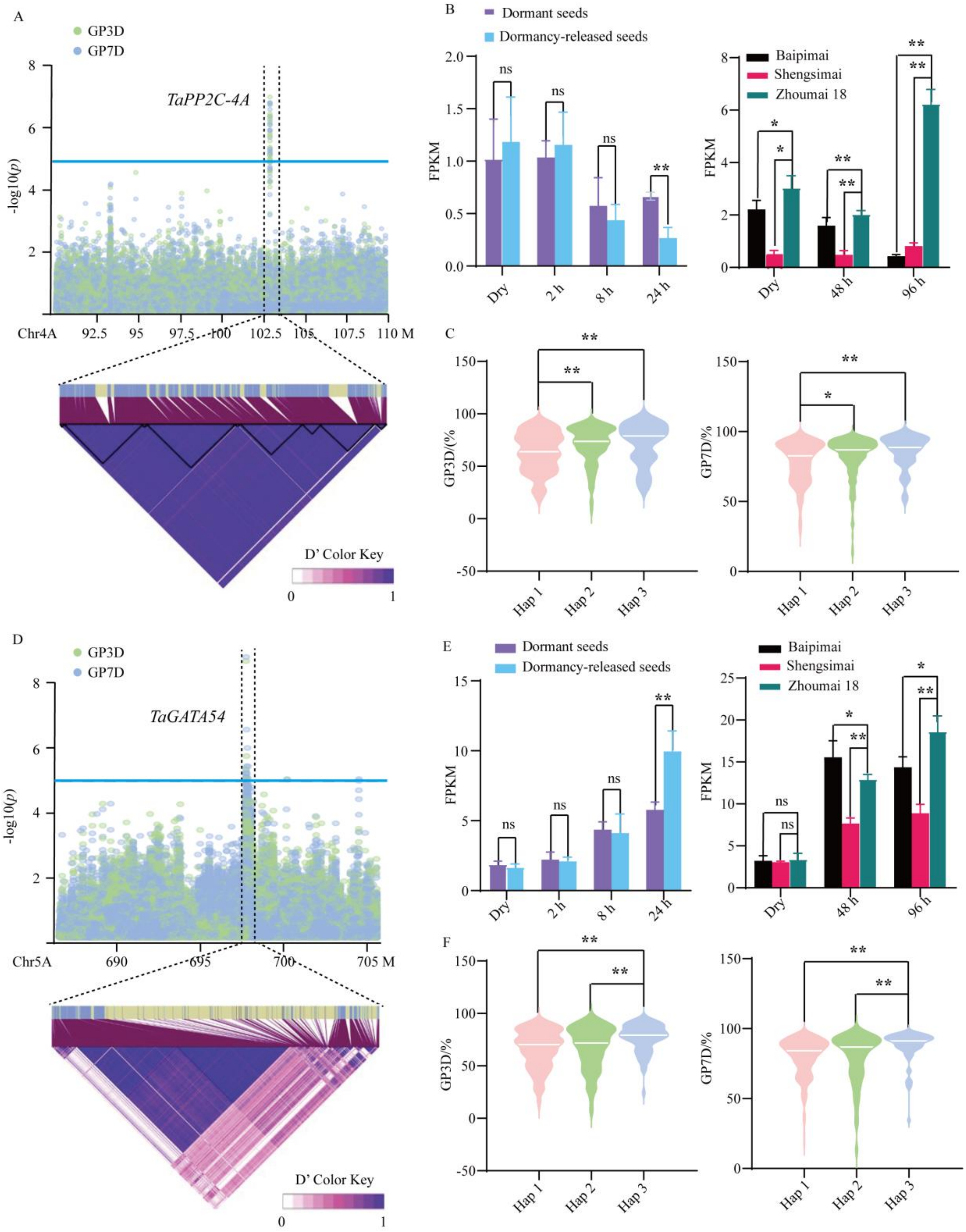

Fig. S4 *TaPP2C-4A* and *TaGATA54* were associated with seed dormancy in wheat. (A and D) Manhattan plots and pairwise LD analysis showing the association SNPs marker and seed dormancy trait on chromosome 4A (A) and 5A (D). Blue line indicated the significance threshold (-log[*P*-value]>5). (B and E) RNA-seq data of *TaPP2C-4A* (B) and *TaGATA54* (E) transcription in different dormancy released seeds from Darius and different dormancy level seeds of wheat varieties (strong dormancy: Baipimai and Shengsimai; weak dormancy: Zhoumai 18) at different germination time points. (C and F) Germination phenotypic difference between three haplotypes for *TaPP2C-4A* (C) and *TaGATA54* (F). Values are means ± SE. Significant differences were determined by Student's *t*-test. *, 0.01<*P* <0.05; **, *P*<0.01.

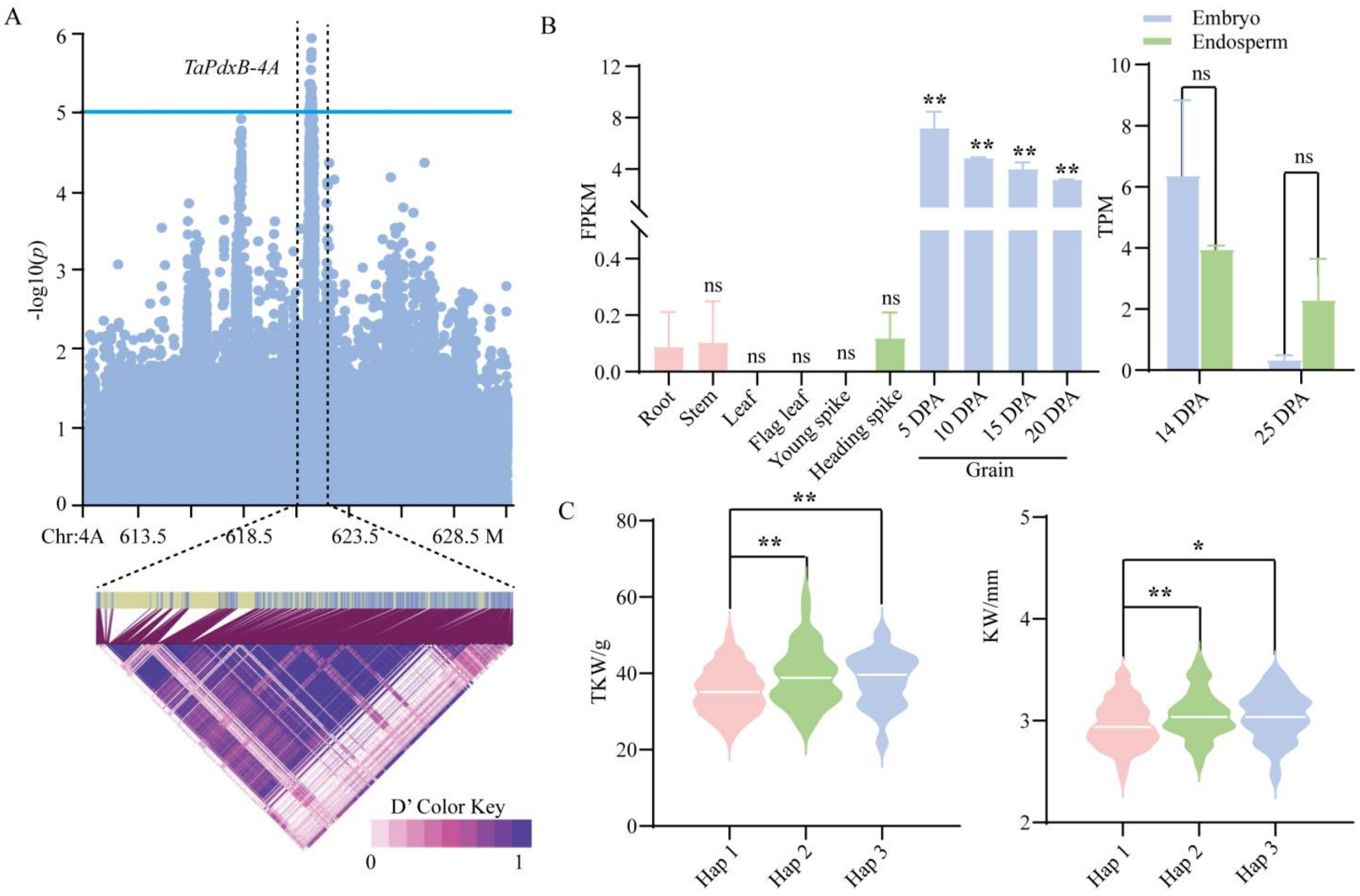

Fig. S5 *TaPdxB-4A* was associated with seed size in wheat. (A) Manhattan plots and pairwise LD analysis showing the association SNPs marker and seed size trait on chromosome 4A. Blue line indicated the significance threshold (-log[*P*-value]>5). (B) RNA-seq data of *TaPdxB-4A* transcription in root, stem, leaf, spike and seed. DPA indicated the day post-anthesis. Values are means ± SE. (C) Seed size phenotypic difference between three haplotypes for *TaPdxB-4A*. Significant differences were determined by Student's *t*-test. *, 0.01<*P*<0.05; **, *P*<0.01.

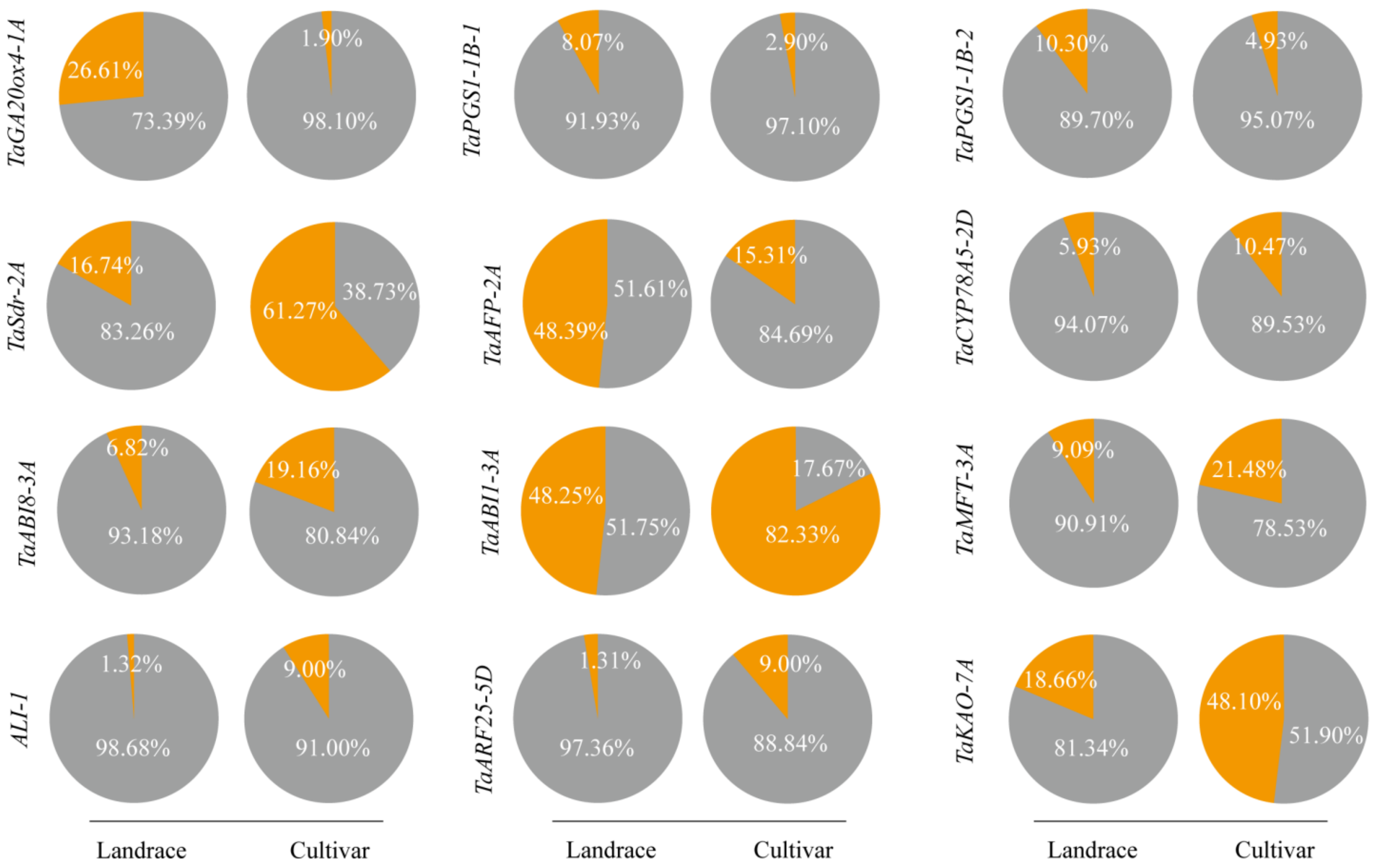

Fig. S6 The comparison of two major haplotypes frequency between landraces and cultivars for seed dormancy/size genes targeted by selection. The haplotype with higher frequency in landrace is designated as Hap 1 (gray) and the lower as Hap 2 (orange).

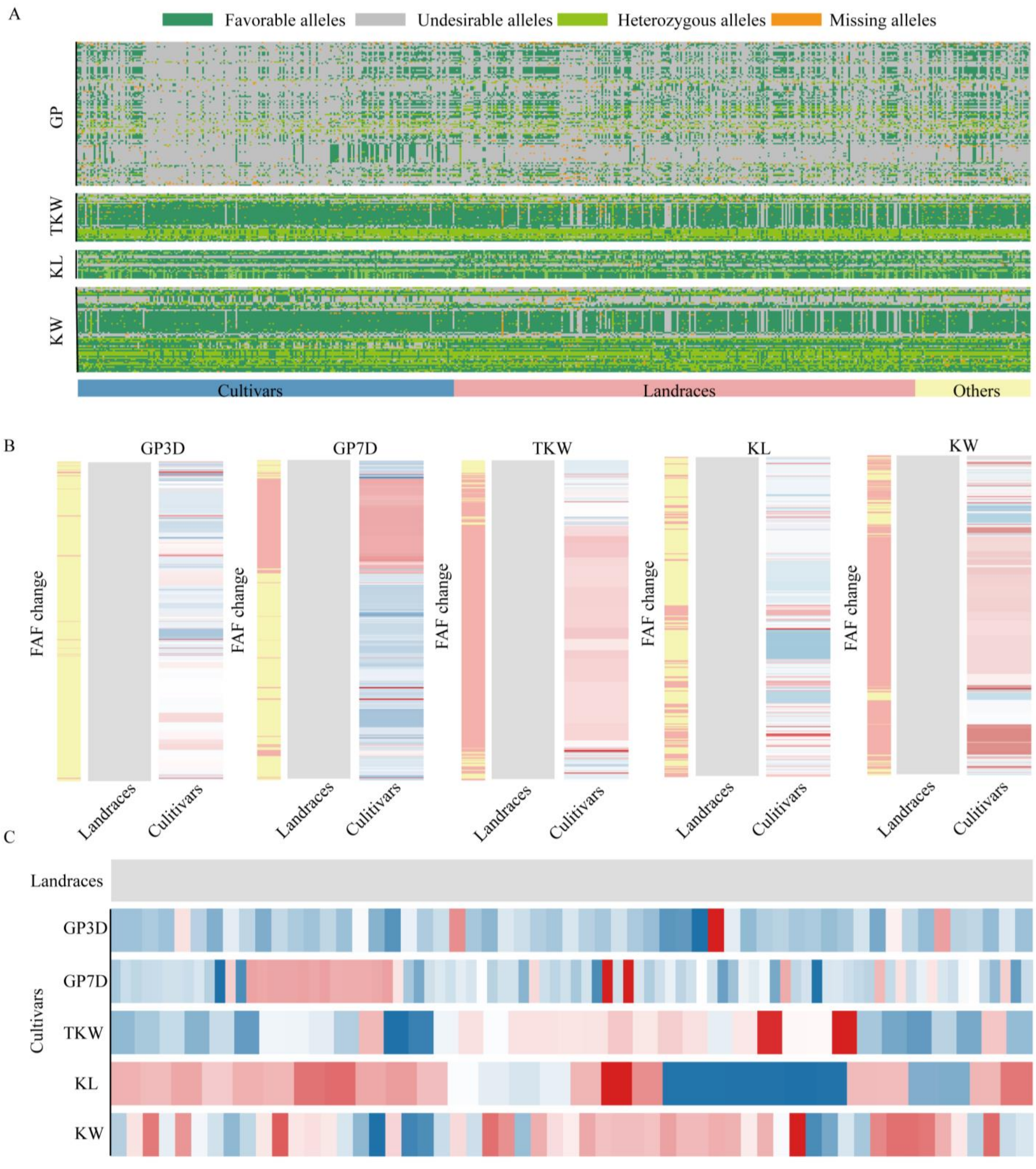

Fig. S7 Breeding selection of favorable alleles and change of FAF. (A) The genomic fingerprints of the 545 common wheat accessions for 229 lead SNPs associated with seed dormancy and seed size traits (each row indicating a lead SNP and each column indicating an accession). Lead SNPs associated with the same trait are grouped together and the trait names are marked in the left of the plot. (B-C) Heatmap of changes in FAF for MTAs (B) and lead SNPs (C) between landraces and cultivars. The pink and yellow colors in the first column stand for the MTA SNPs and the remaining SNPs (-log[*P*-value]<5). Red represents an increase in FAF, blue represents a decrease, gray represents no change in FAF. Each row or column stands for an associated SNP.

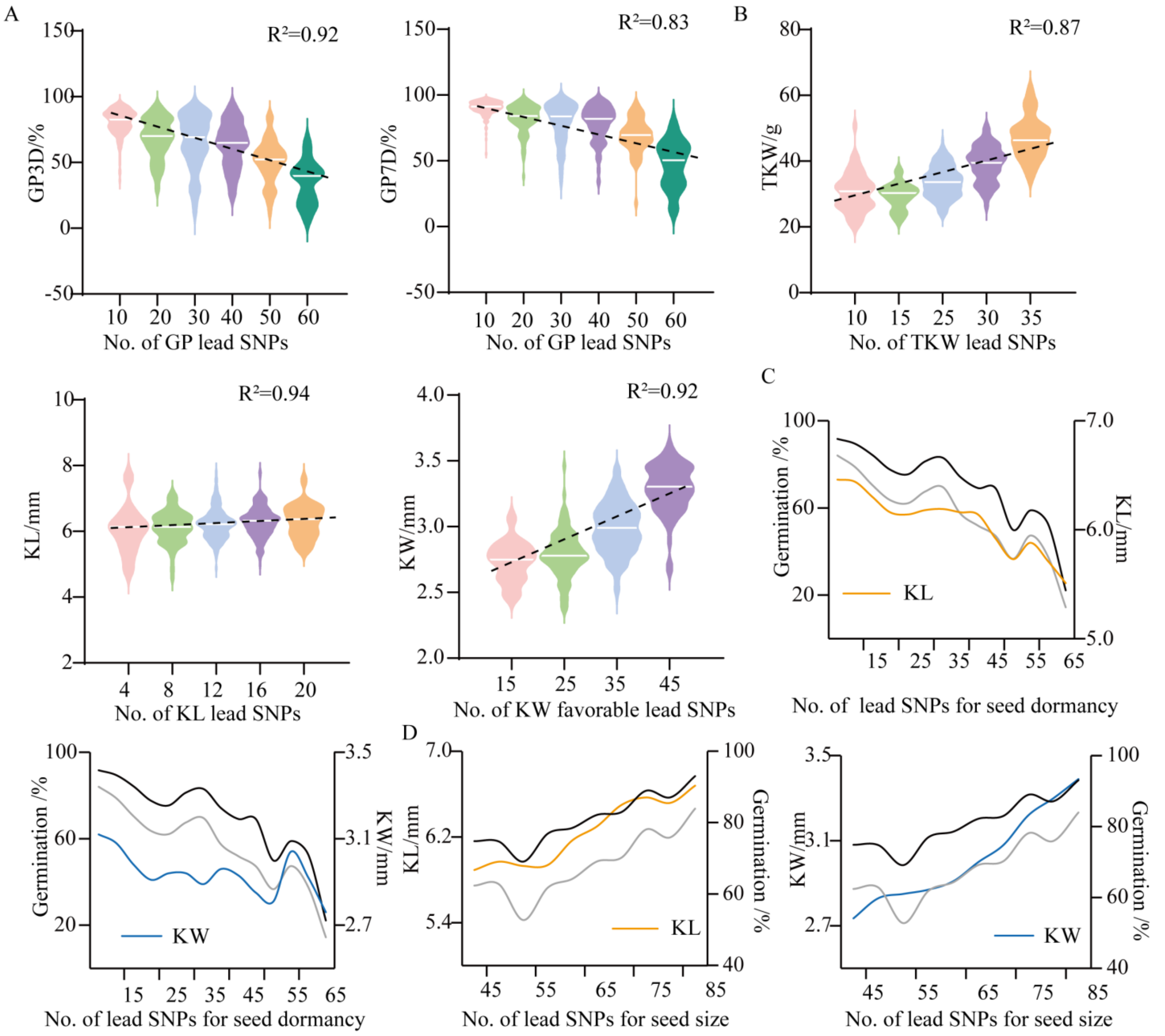

Fig. S8 Relationships between seed dormancy or seed size favorable allele number and phenotypic values. (A) The relationships between the number of seed dormancy favorable alleles and phenotypic values for GP3D, GP7D. (B) The relationships between the number of seed size favorable alleles and phenotypic values for TKW, KL, and KW. R^2 means the correlation coefficient of phenotypic and numbers of favorable alleles. (C) Change trend plots indicating the negative correlation between number of favorable alleles of seed dormancy and seed size trait. (D) Change trend plots indicating the negative correlation between number of favorable alleles of seed size and seed dormancy trait. The gray, black, orange and blue lines represent GP3D, GP7D, KL, and KW.

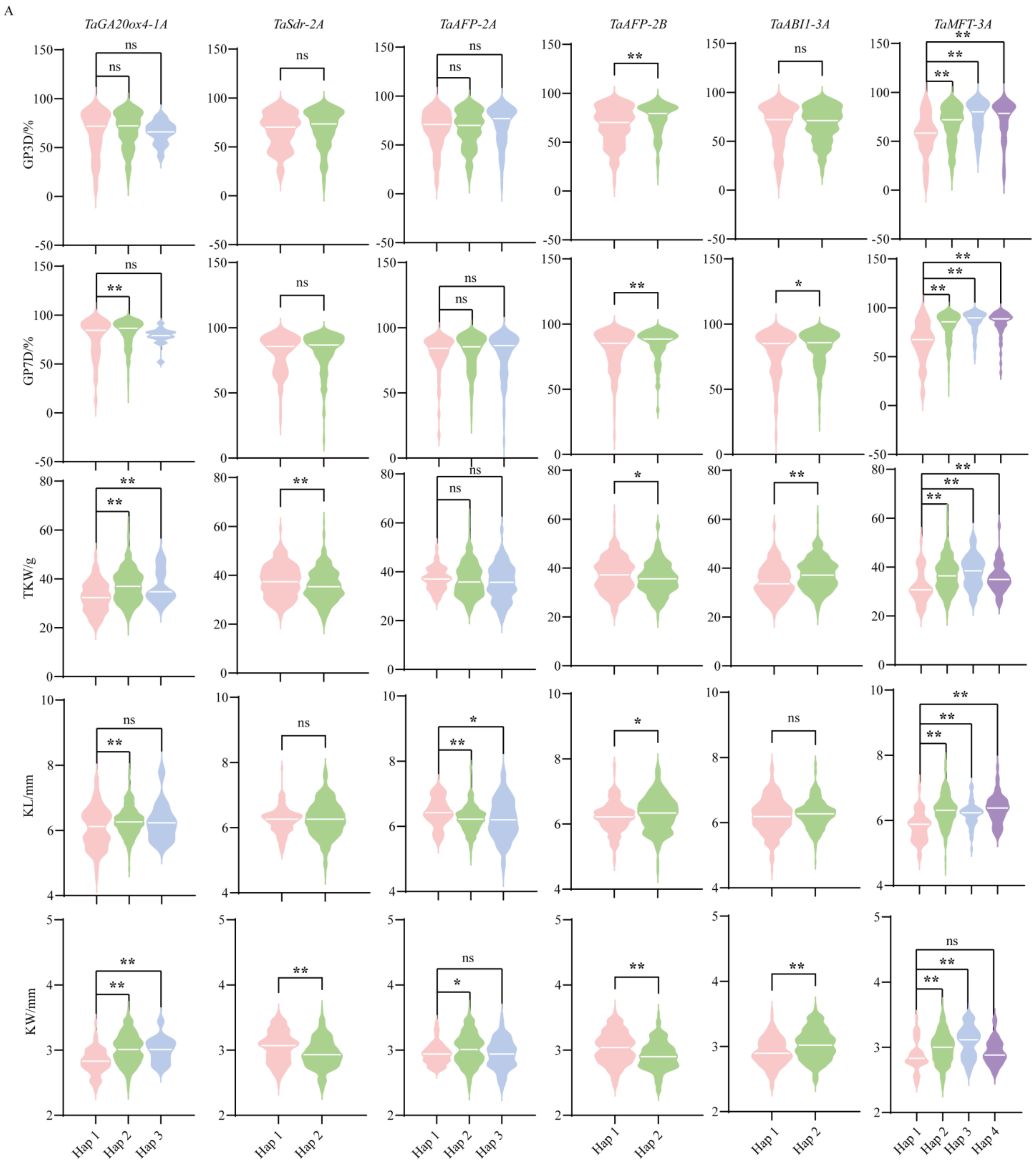

Fig. S9A Phenotypic difference between haplotypes for known seed dormancy genes. (A) Haplotype analysis for *TaGA20ox4-1A*, *TaSdr-2A*, *TaAFP-2A*, *TaAFP-2B*, *TaABI1-3A* and *TaMFT-3A*. Significant differences were determined by Student's *t*-test. *, 0.01<*P*<0.05; **, *P*<0.01.

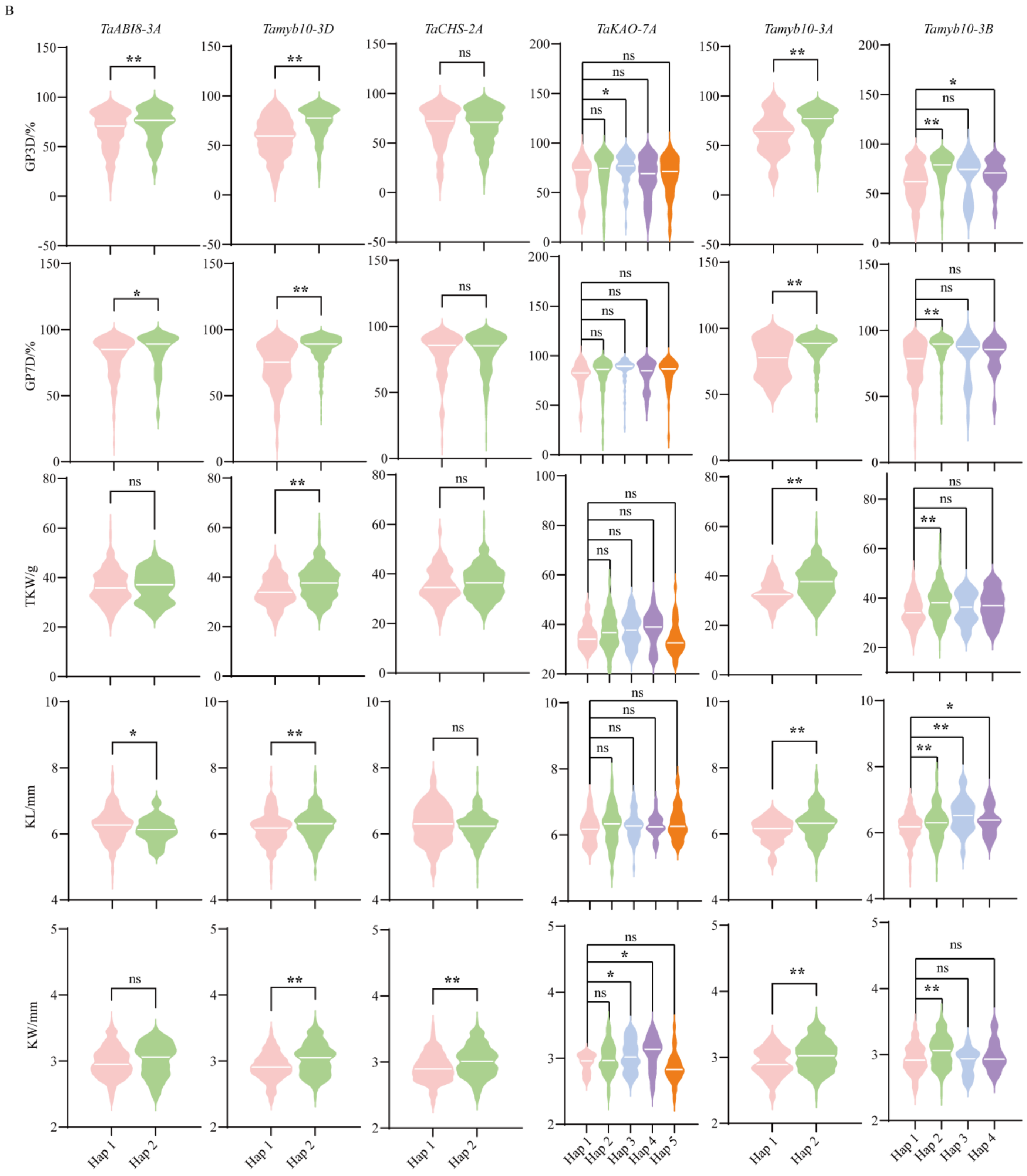

Fig. S9B Phenotypic difference between haplotypes for known seed dormancy genes. (B) Haplotype analysis for *TaABI8-3A*, *Tamyb10-3D*, *TaCHS-2A*, *TaKAO-7A*, *Tamyb10-3A* and *Tamyb10-3B*. Significant differences were determined by Student's *t*-test. *, 0.01<*P*<0.05; **, *P*<0.01.

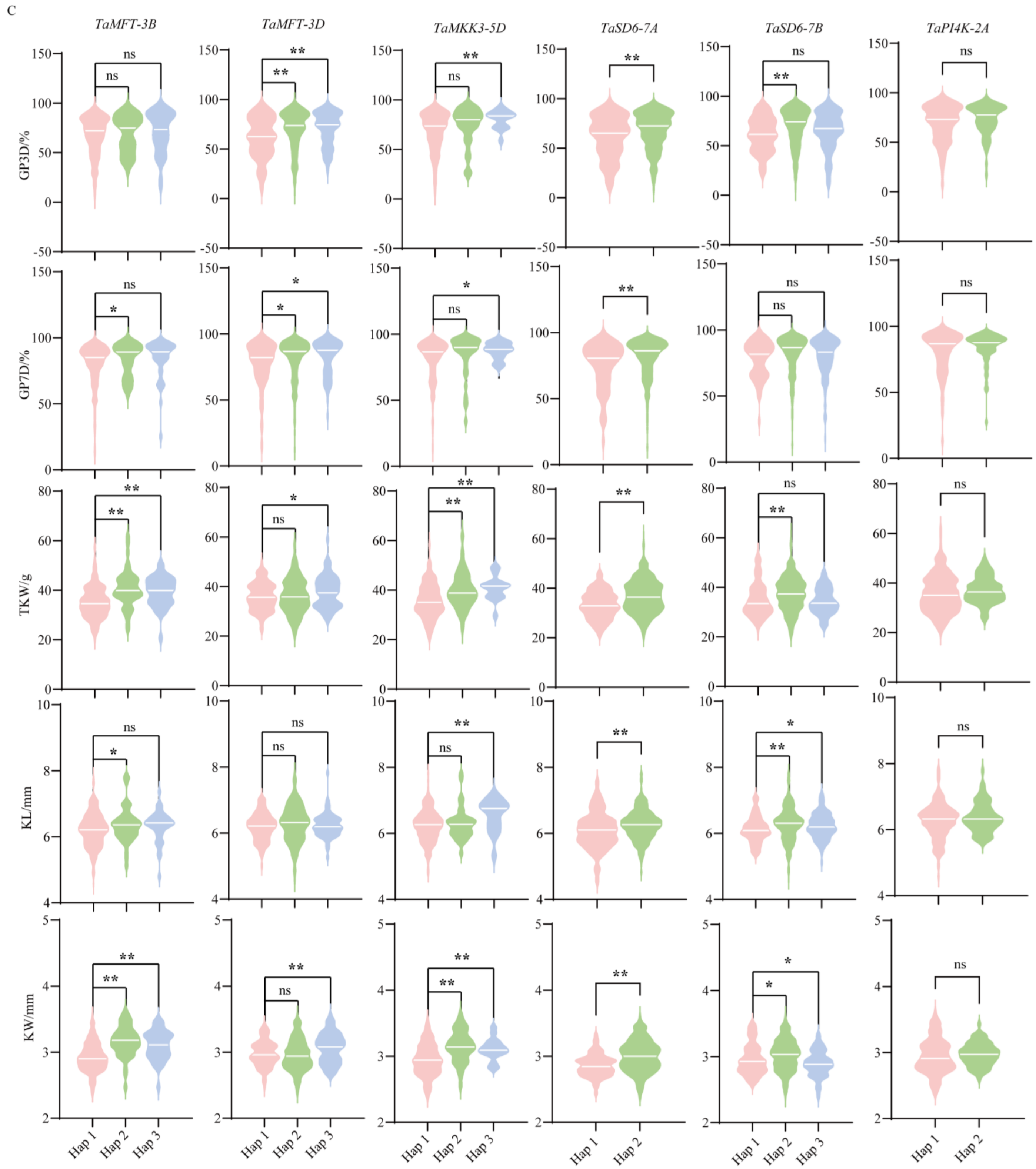

Fig. S9C Phenotypic difference between haplotypes for known seed dormancy genes. (C) Haplotype analysis for *TaMFT-3B*, *TaMFT-3D*, *TaMKK3-5D*, *TaSD6-7A*, *TaSD6-7B* and *TaPI4K-2A*. Significant differences were determined by Student's *t*-test. *, 0.01<P<0.05; **, P<0.01.

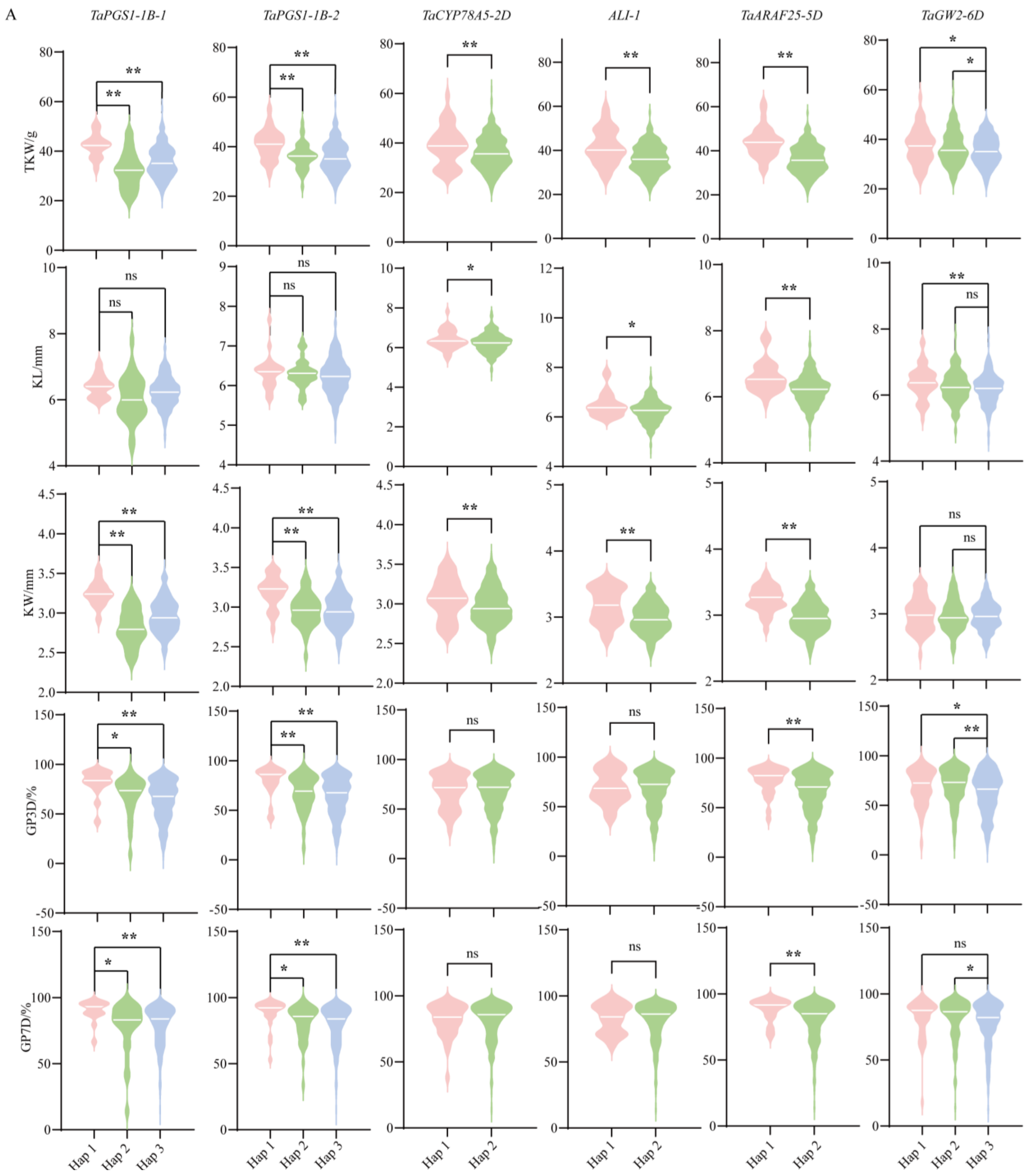

Fig. S10A Phenotypic difference between haplotypes for known seed size genes. (A) Haplotype analysis for *TaPGSI-1B-1*, *TaPGSI-1B-2*, *TaCYP78A5-2D*, *ALI-1*, *TaARAF25-5D* and *TaGW2-6D*. Significant differences were determined by Student's *t*-test. *, 0.01<*P*<0.05; **, *P*<0.01.

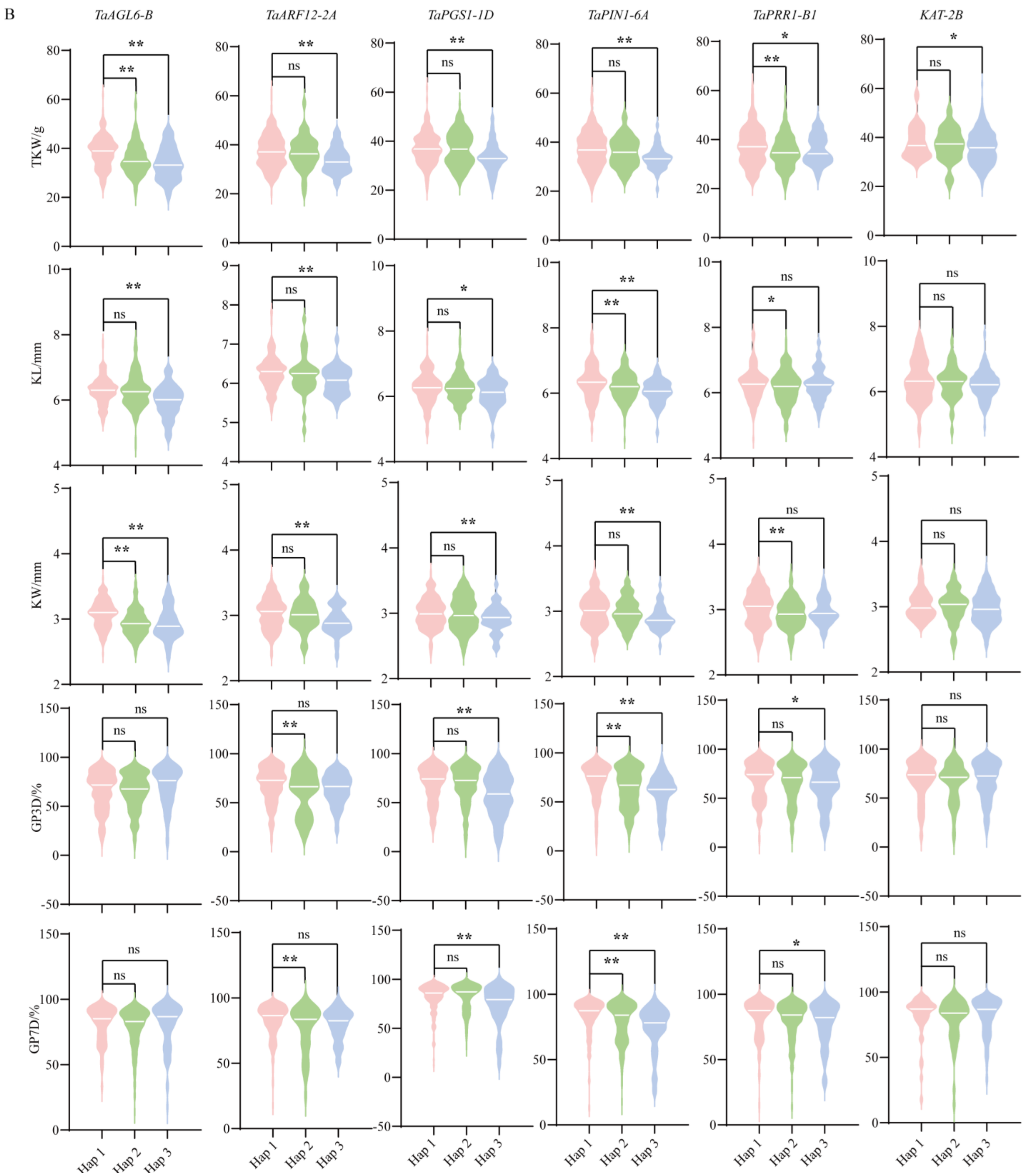

Fig. S10B Phenotypic difference between haplotypes for known seed size genes. (B) Haplotype analysis for *TaAGL6-B*, *TaAFR12-2A*, *TaPGS1-1D*, *TaPIN1-6A*, *TaPRR1-B1* and *KAT-2B*. Significant differences were determined by Student's *t*-test. *, 0.01<*P*<0.05; **, *P*<0.01.

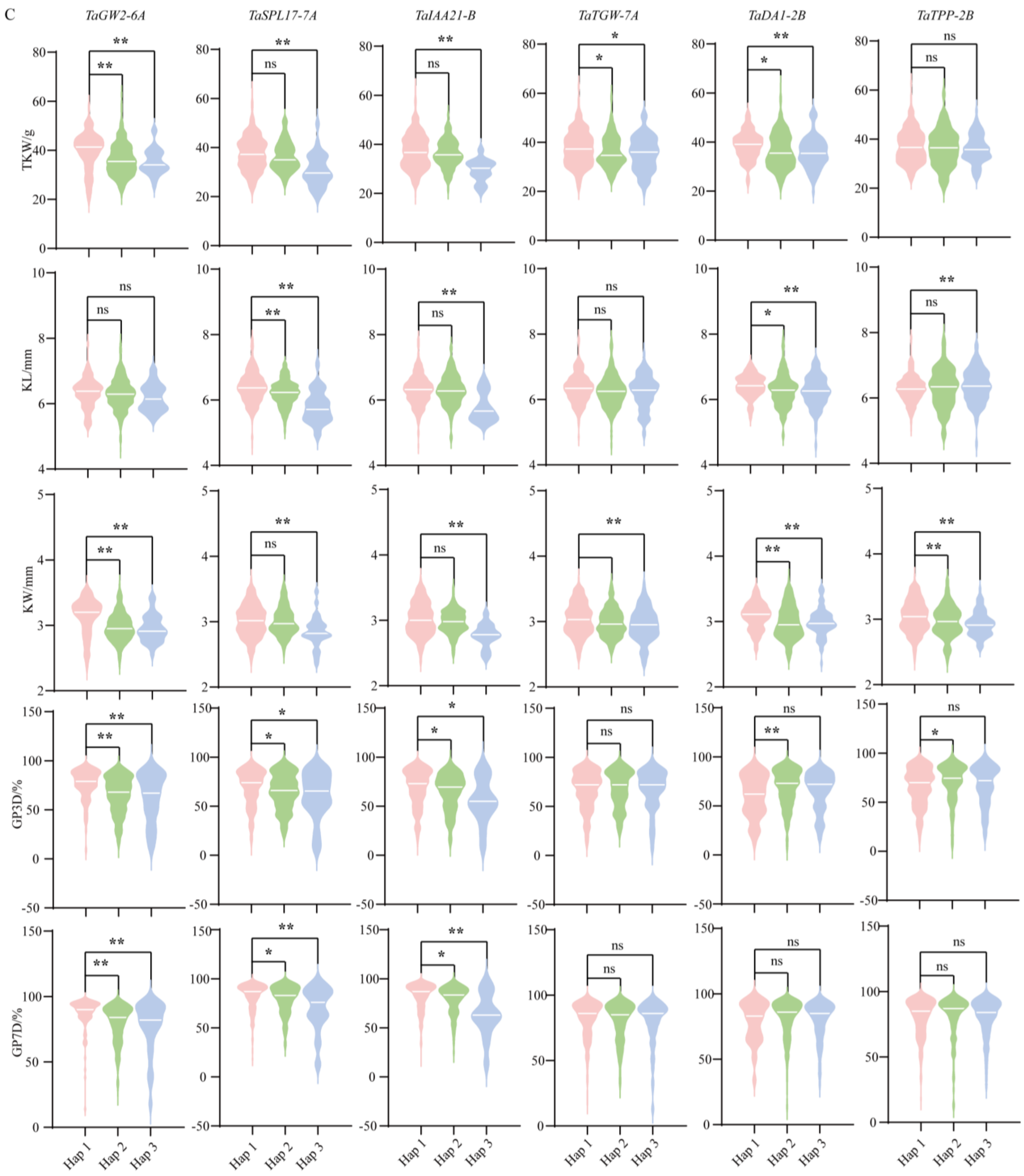

Fig. S10C Phenotypic difference between haplotypes for known seed size genes. (C) Haplotype analysis for *TaGW2-6A*, *TaSPL17-7A*, *TaIAA21-B*, *TaTGW-7A*, *TaDA1-2B* and *TaTPP-2B*. Significant differences were determined by Student's *t*-test. *, 0.01<P<0.05; **, P<0.01.

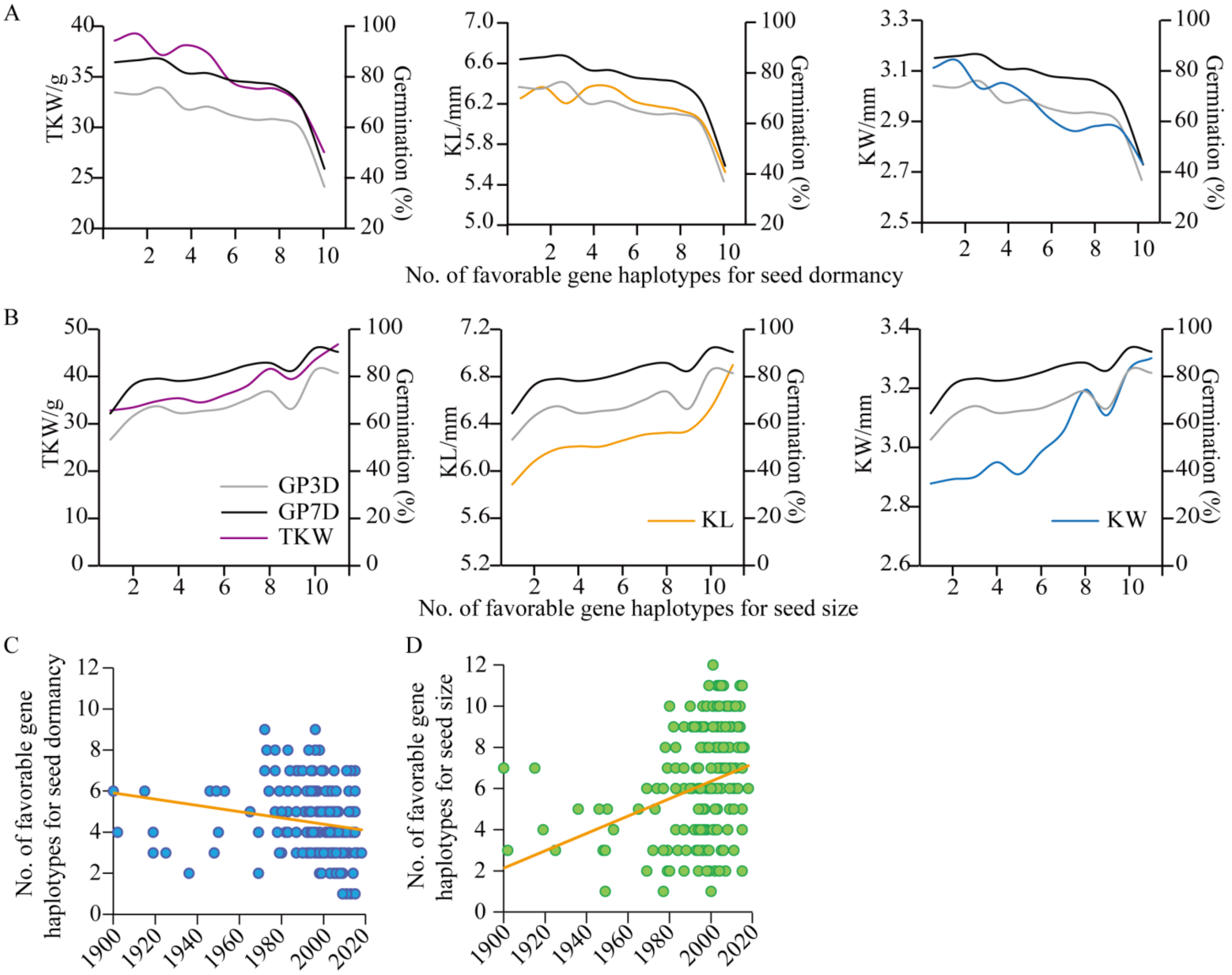

Fig. S11 The relationships between number of favorable haplotypes and seed dormancy trait or seed size trait. (A) Relationships between the number of favorable haplotypes of seed dormancy genes indicated in Fig. S9 and seed size traits. (B) Relationships between the number of favorable haplotypes of seed size genes indicated in Fig. S10 and seed dormancy traits. The number change of favorable alleles of seed dormancy (C) and seed size (D) in different released years for cultivars.

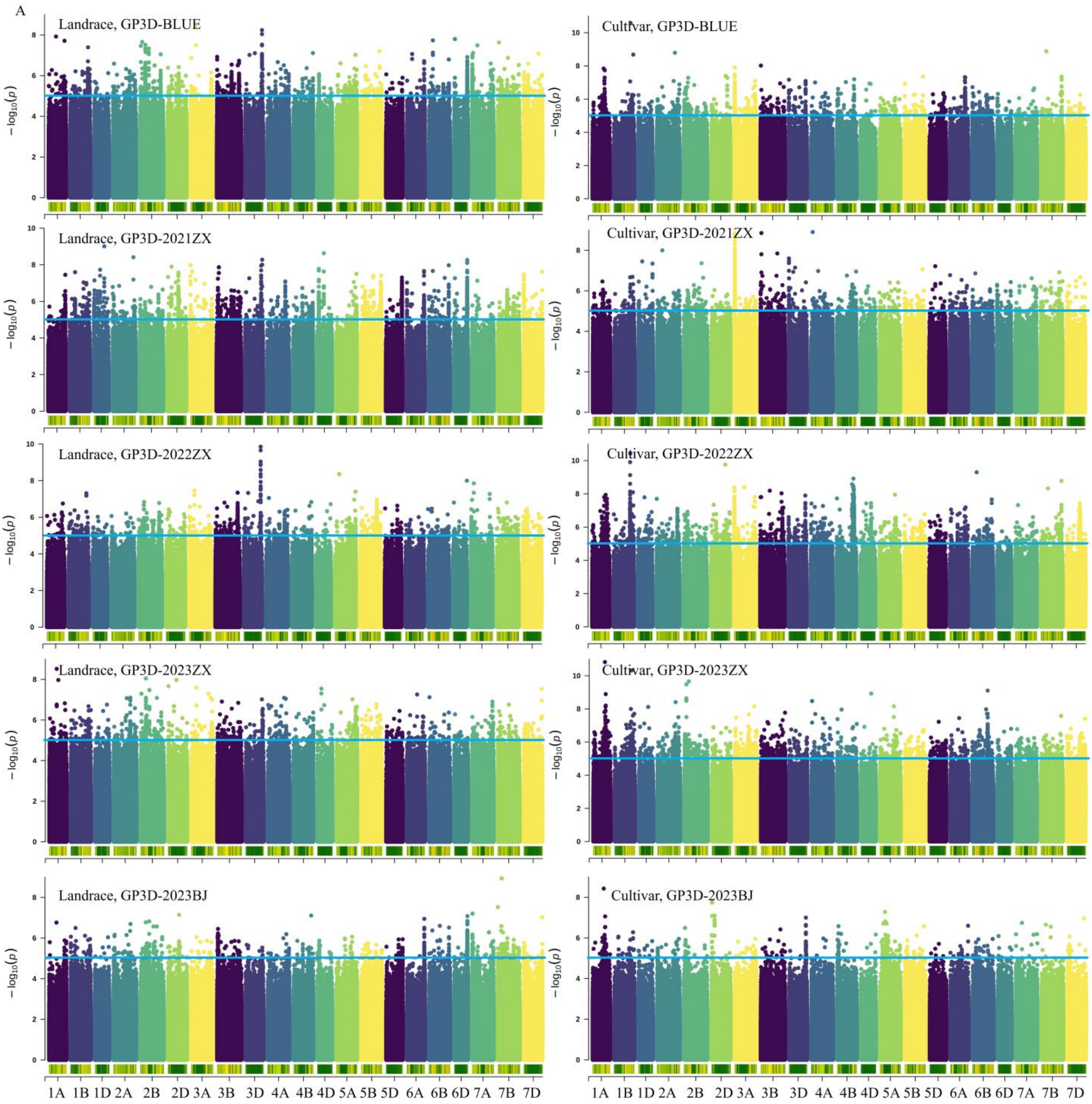

Fig. S12A Genome-wide association analysis of seed dormancy and seed size using landrace (left panel) and cultivar (right panel) wheat accessions. (A) Manhattan plots showing the SNP marker-trait associations for GP3D. Blue line indicated the significance threshold (-log[*P*-value]>5).

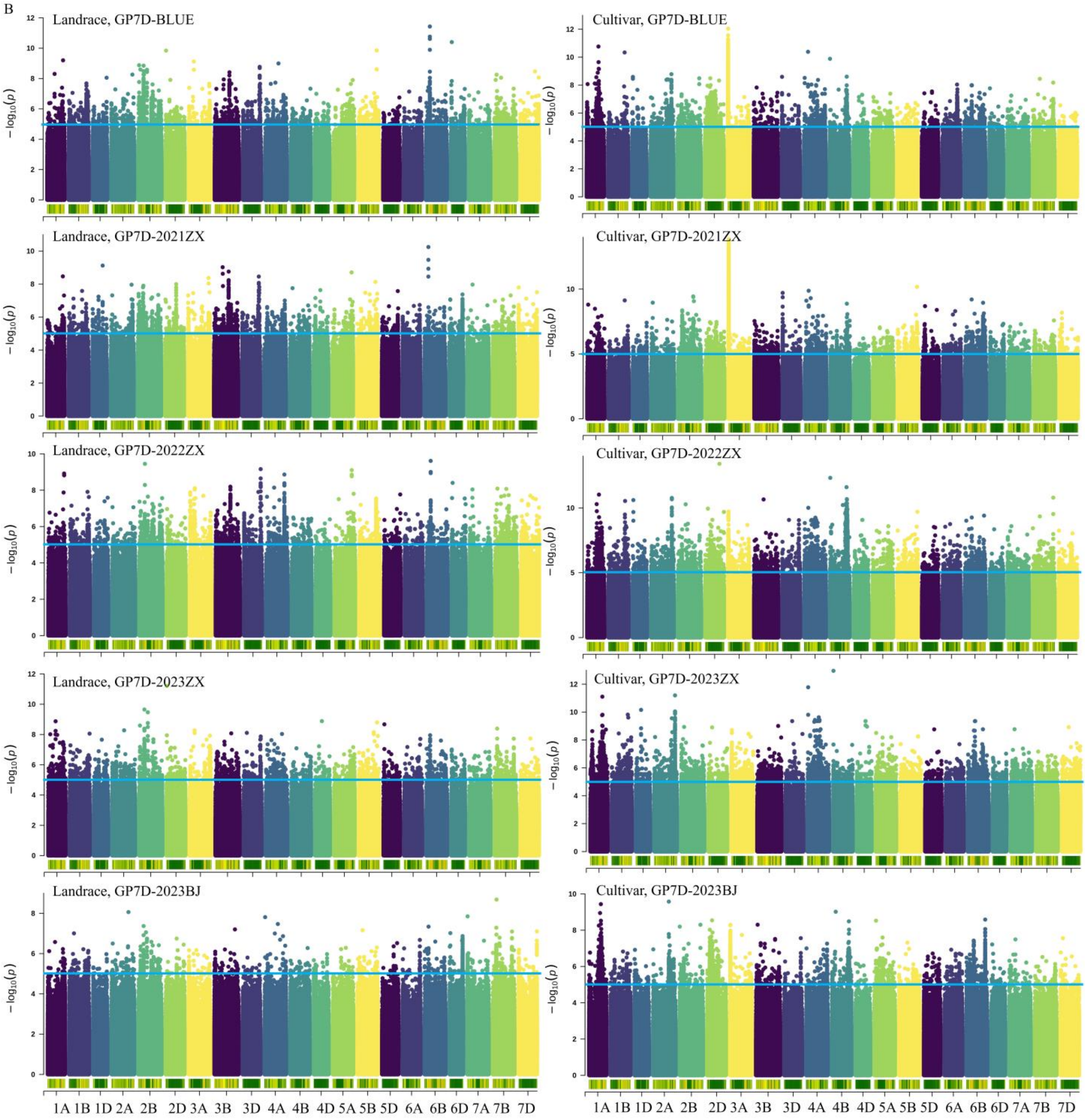

Fig. S12B Genome-wide association analysis of seed dormancy and seed size using landrace (left panel) and cultivar (right panel) wheat accessions. (B) Manhattan plots showing the SNP marker-trait associations for GP7D. Blue line indicated the significance threshold (-log[*P*-value]>5).

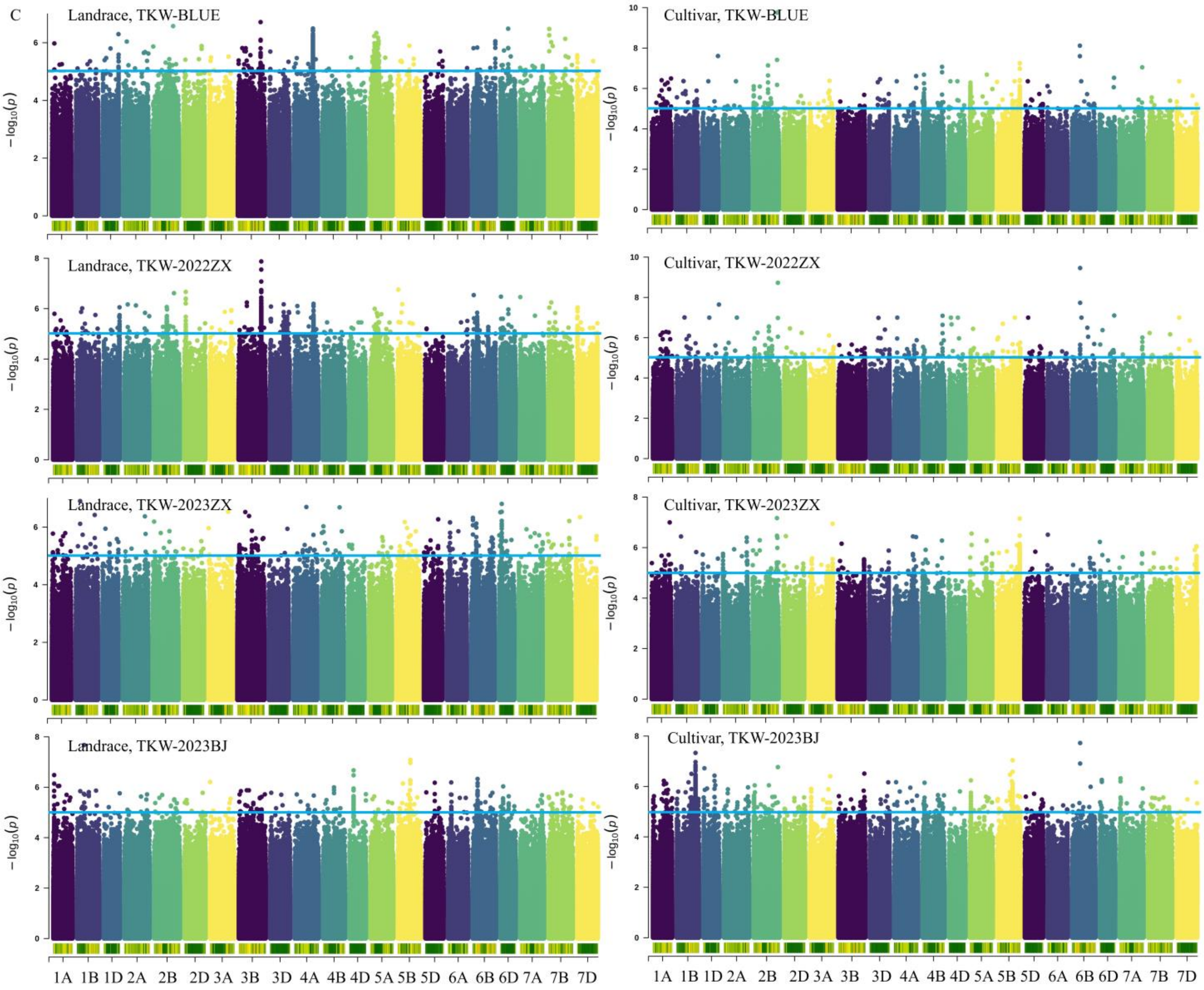

Fig. S12C Genome-wide association analysis of seed dormancy and seed size using landrace (left panel) and cultivar (right panel) wheat accessions. (C) Manhattan plots showing the SNP marker-trait associations for TKW. Blue line indicated the significance threshold (-log[*P*-value]>5).

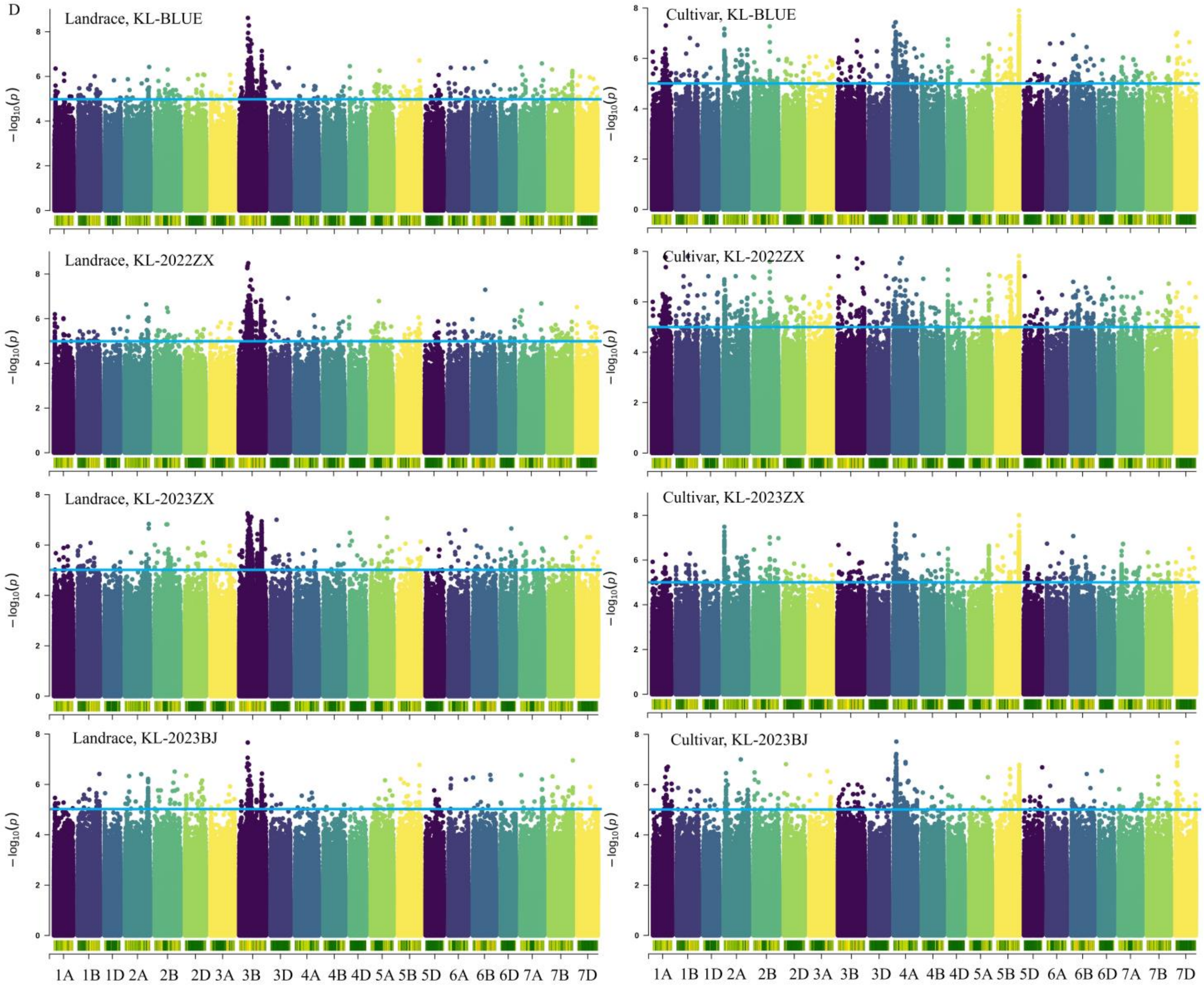

Fig. S12D Genome-wide association analysis of seed dormancy and seed size using landrace (left panel) and cultivar (right panel) wheat accessions. (D) Manhattan plots showing the SNP marker-trait associations for KL. Blue line indicated the significance threshold (-log[*P*-value]>5).

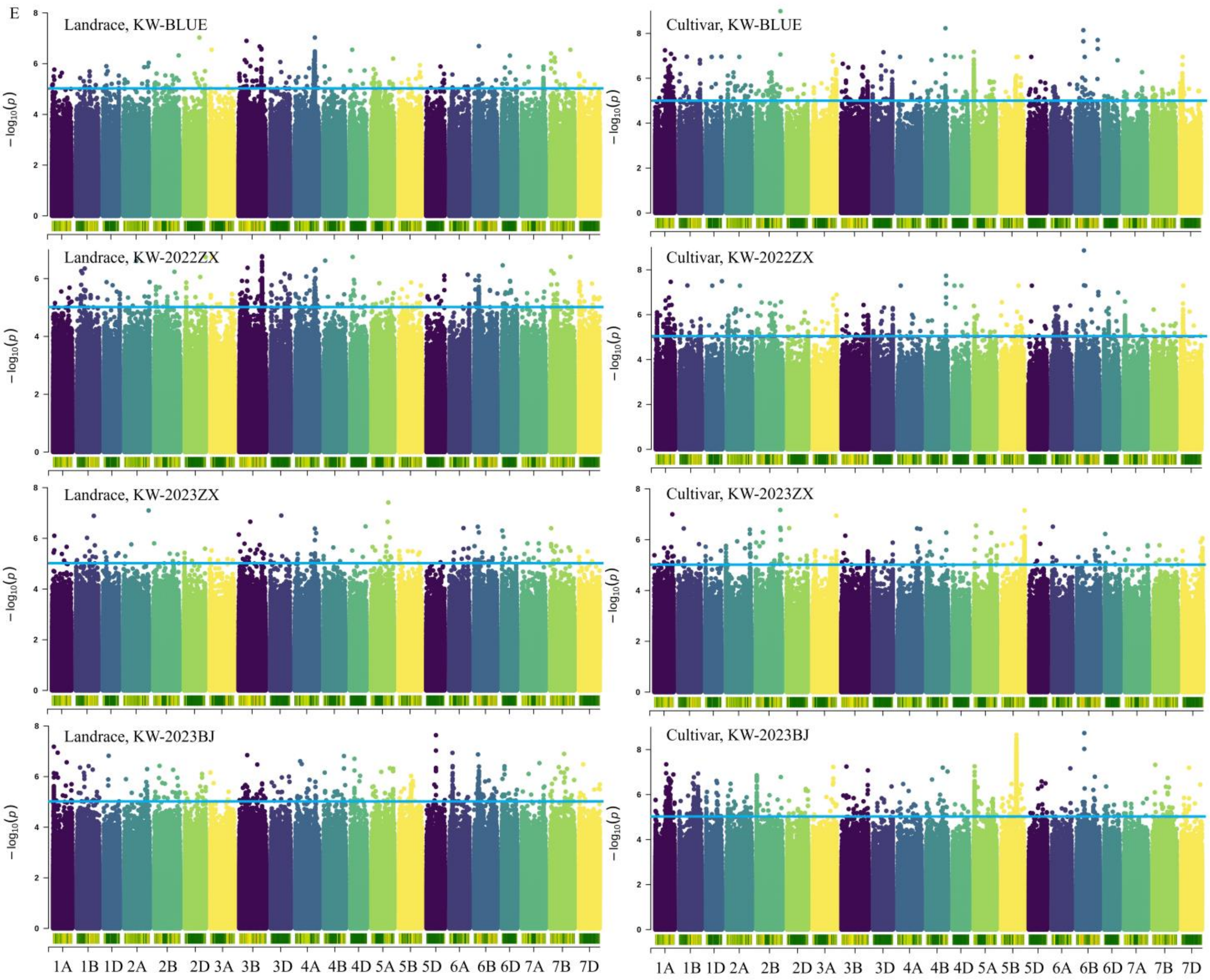

Fig. S12E Genome-wide association analysis of seed dormancy and seed size using landrace (left panel) and cultivar (right panel) wheat accessions. (E) Manhattan plots showing the SNP marker-trait associations for KW. Blue line indicated the significance threshold (-log[*P*-value]>5).

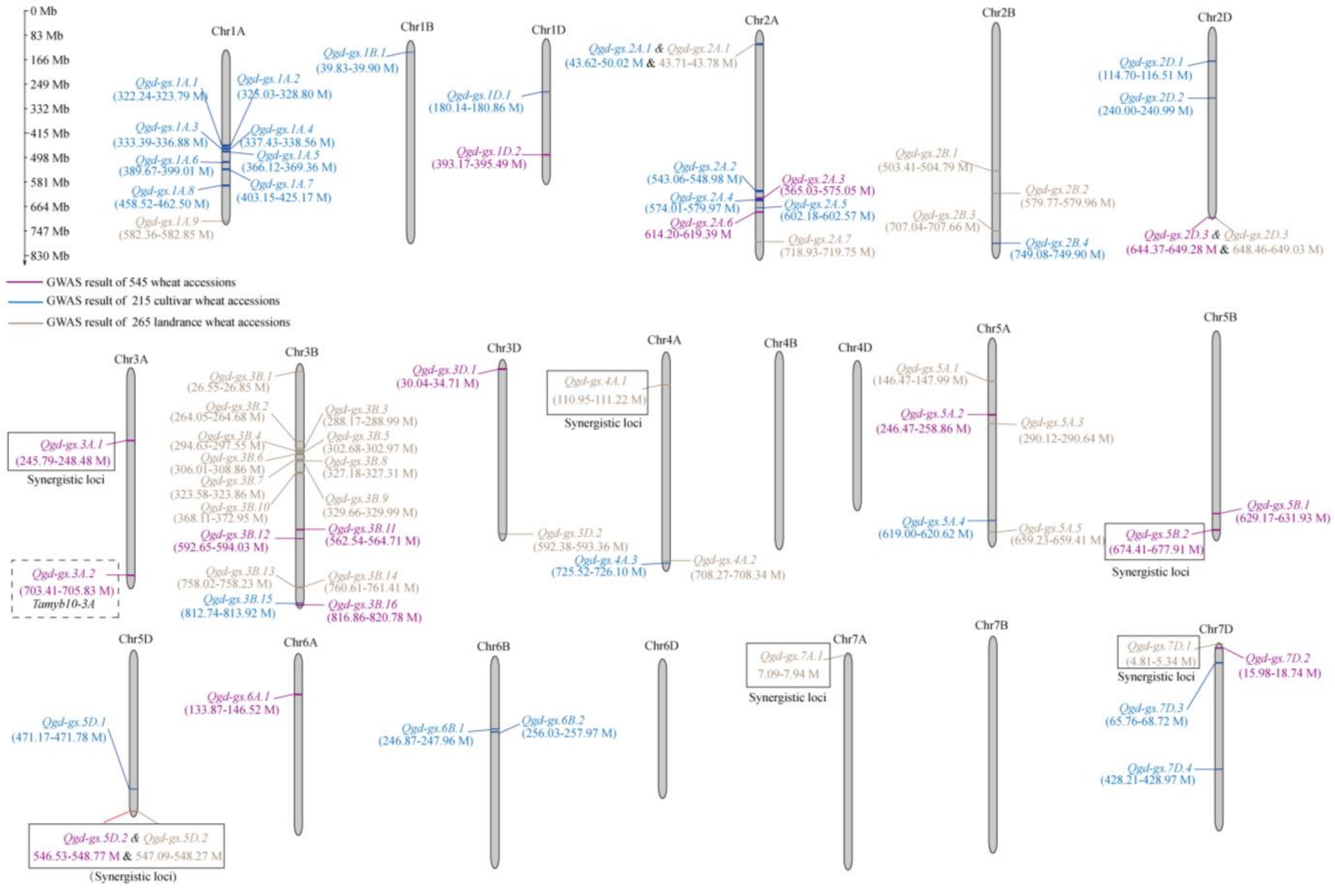

Fig. S13 The overlapping regions between seed dormancy and seed size were identified by GWAS result. The purple, blue and brown lines represent GWAS result of 545 wheat accessions, 215 cultivar wheat accessions and 265 landrace wheat accessions, respectively.

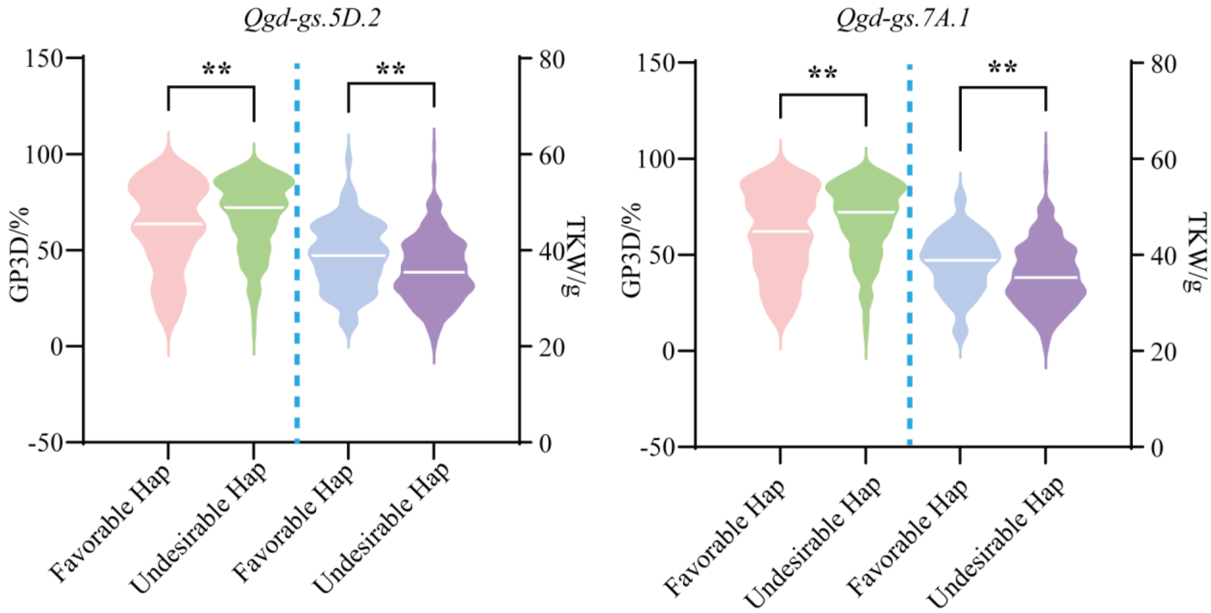

Fig. S14 GP3D and TKW difference between favorable and undesirable haplotype for *Qgd-gs.5D.2* and *Qgd-gs.7A.1*. Significant differences were determined by Student's *t*-test. *, $0.01<P<0.05$; **, $P<0.01$.

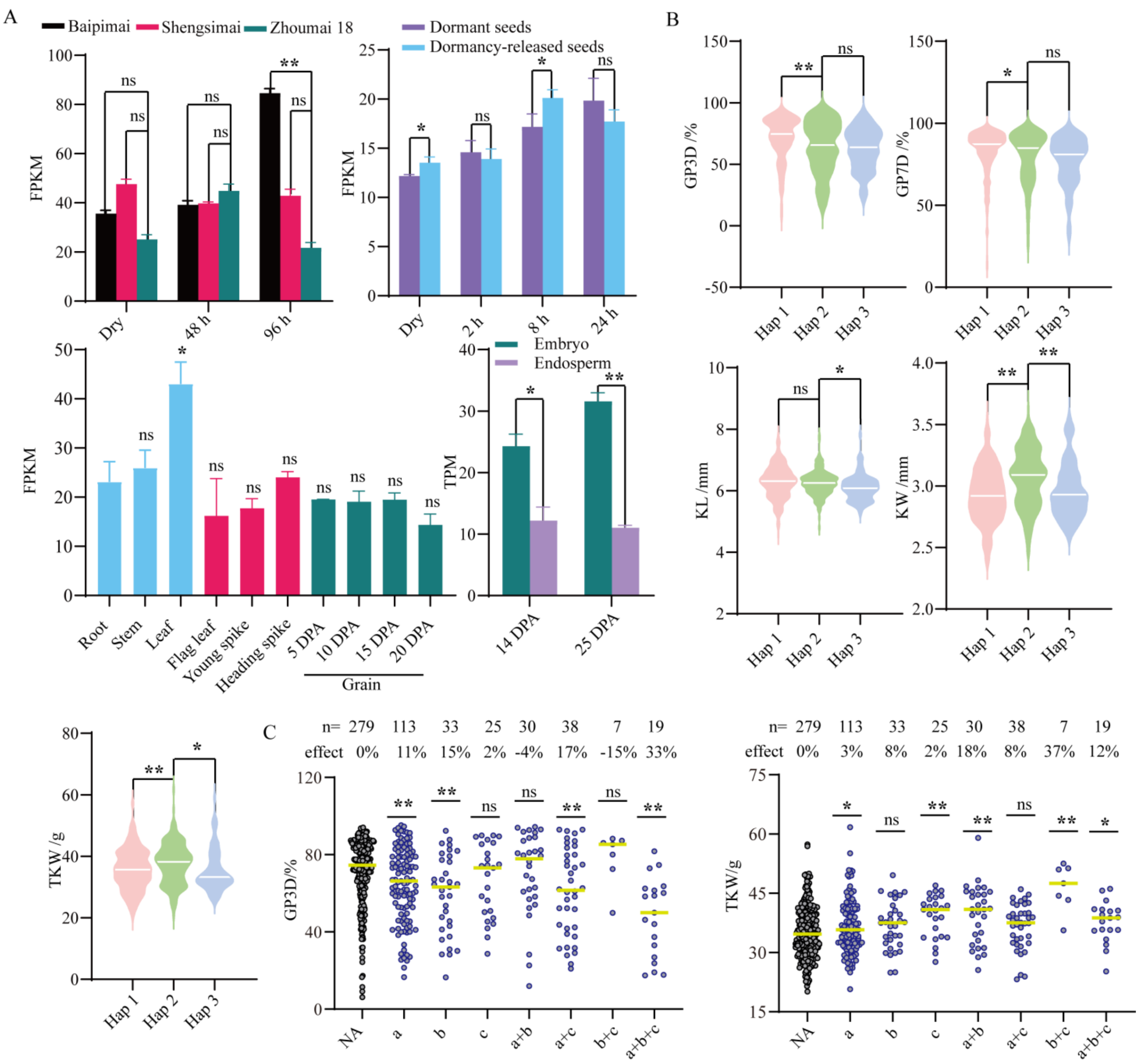

Fig. S15 *TaRBP-4A* was associated with seed dormancy and seed size in wheat and the phenotypic effects of allelic combinations of *Qgd-gs.4A.1, Qgd-gs.5D.2 and Qgd-gs.7A.1* for GP3D and TKW in the 545 wheat accessions collection. (A) RNA-seq data of *TaRBP-4A* transcription in different dormancy released seeds from Darius and different dormancy level seeds of wheat varieties at different germination time points (upper panel). RNA-seq data of *TaRBP-4A* transcription in root, stem, leaf, spike and seed (lower panel). DPA indicated the day post-anthesis. Values are means ± SE. *, 0.01<P<0.05; **, P<0.01. (B) Seed dormancy and seed size phenotypic difference between three haplotypes for *TaRBP-4A*. (C) Dot plots show the phenotypic effects of allelic combinations of *Qgd-gs.4A.1*, *Qgd-gs.5D.2* and *Qgd-gs.7A.1* for GP3D and TKW in the 545 wheat accessions collection. Blue dots represent phenotypic values of each accession carrying allelic combinations indicated below. Yellow lines are the medians of each category. NA indicates accessions carrying no favorable alleles. “a” represents *Qgd-gs.4A.1*, “b” represents *Qgd-gs.5D.2* and “c” represents *Qgd-gs.7A.1*. Phenotypic effects shown at the top were calculated as described in methods. Significant differences were determined by Student’s *t*-test. *, 0.01<P <0.05; **, P<0.01.

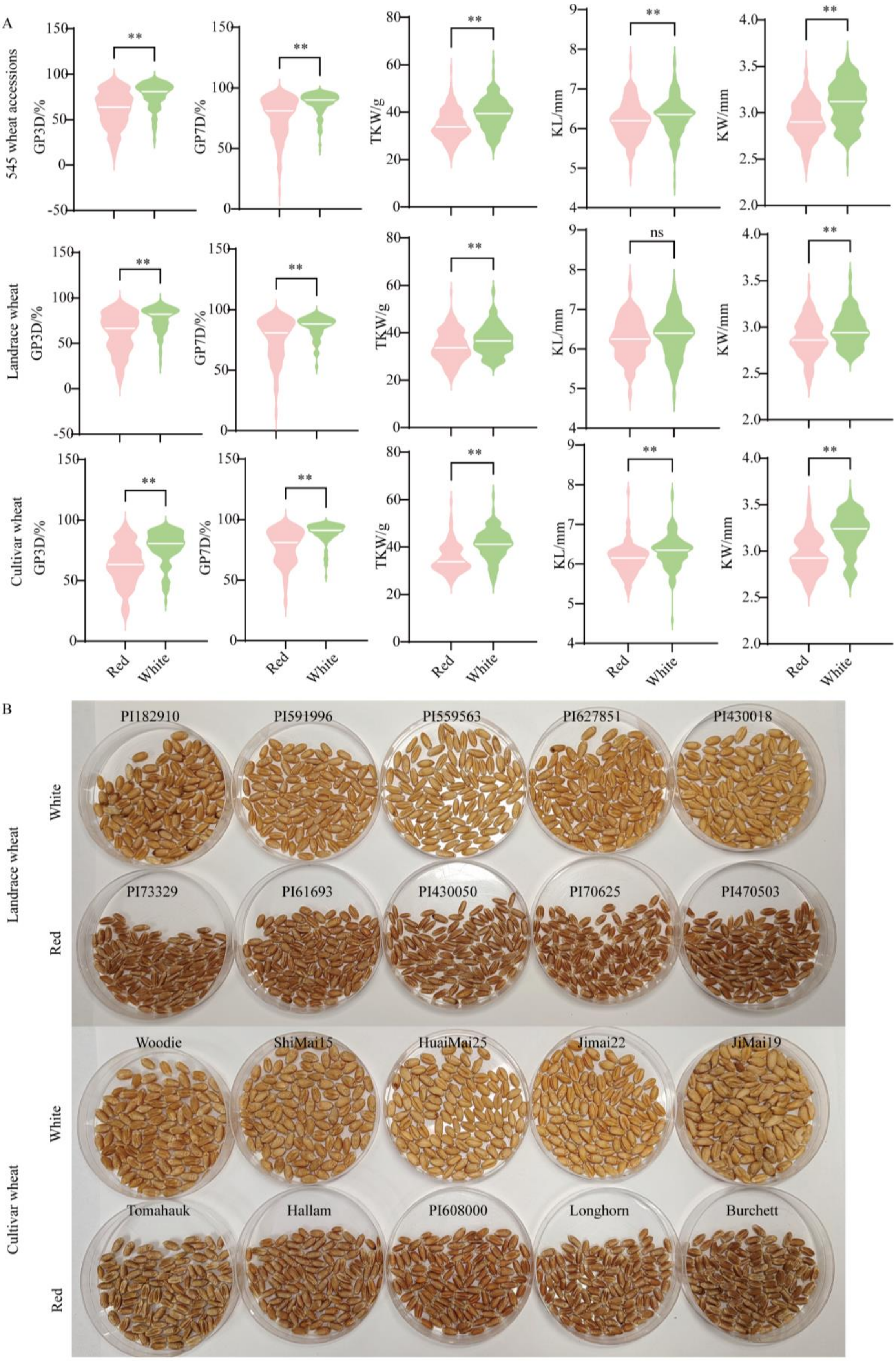

Fig. S16 Correlation analysis between seed color and phenotype in wheat. (A) Correlation analysis of seed color and five phenotypes indexes (GP3D, GP7D, TKW, KL and KW) in 545 wheat accessions, landrace wheat and cultivar wheat. (B) Difference of 100 seeds weight between red seed and white seed wheats with representative germplasm.

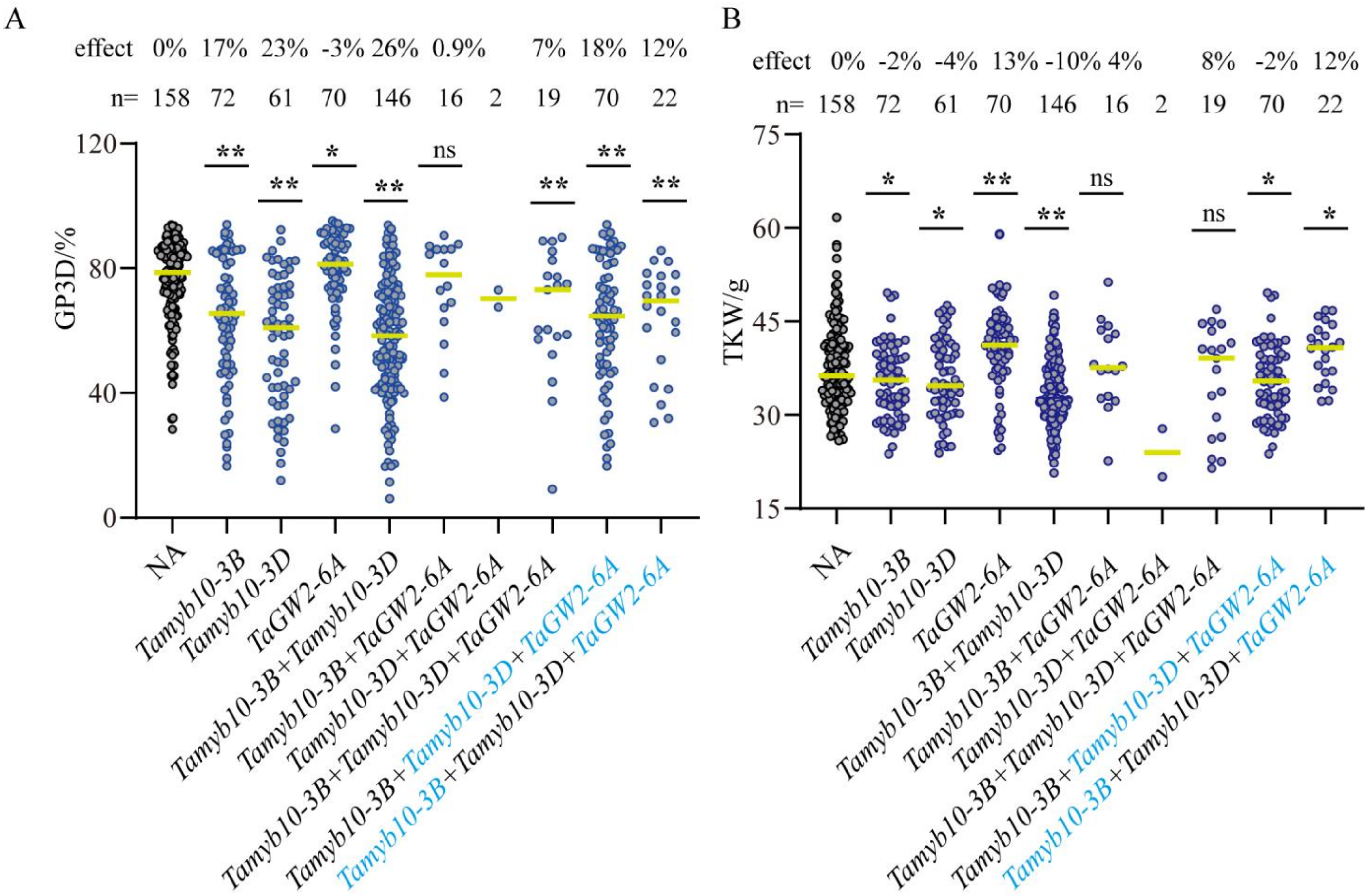

Fig. S17 The phenotypic effects of allelic combinations of *Tamyb10-3B/D* and *TaGW2* for GP3D (A) and TKW (B) in the 545 wheat accessions collection. Blue dots represent phenotypic values of each accession carrying allelic combinations indicated below. Yellow lines are the medians of each category. NA indicates accessions carrying no favorable alleles. Phenotypic effects shown at the top were calculated as described in methods. The blue word represent middle haplotype. Significant differences were determined by Student's *t*-test. *, 0.01<*P*<0.05; **, *P*<0.01.

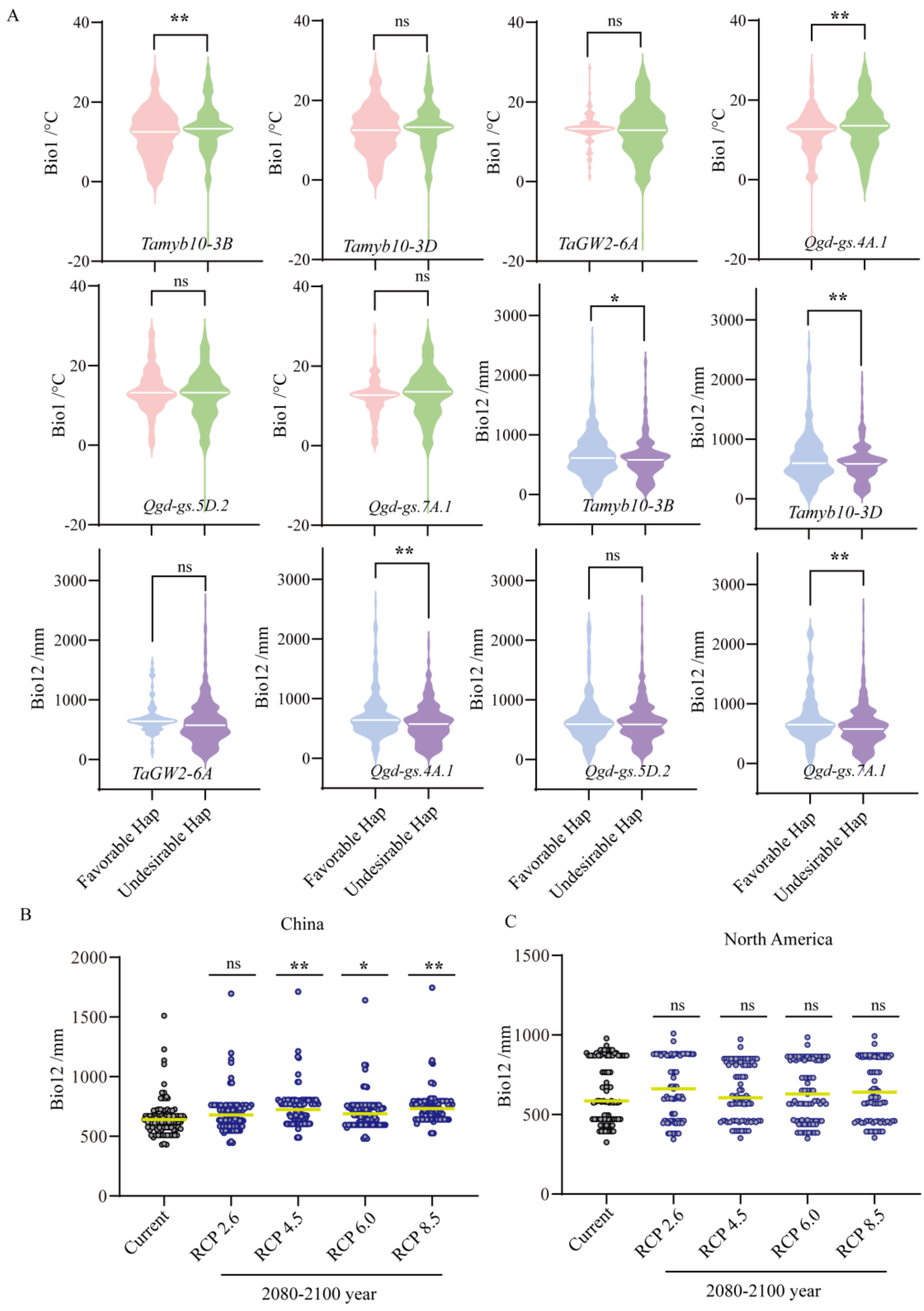

Fig. S18 Comparison of favorable and undesirable haplotypes of pleiotropic genes and synchronous loci for Bio1 and Bio12 (A) and under future (2080-2100 year) climate scenarios, the precipitation in China (B) and North America (C) will increase. Significant differences were determined by Student's *t*-test. *, 0.01<*P*<0.05; **, *P*<0.01.

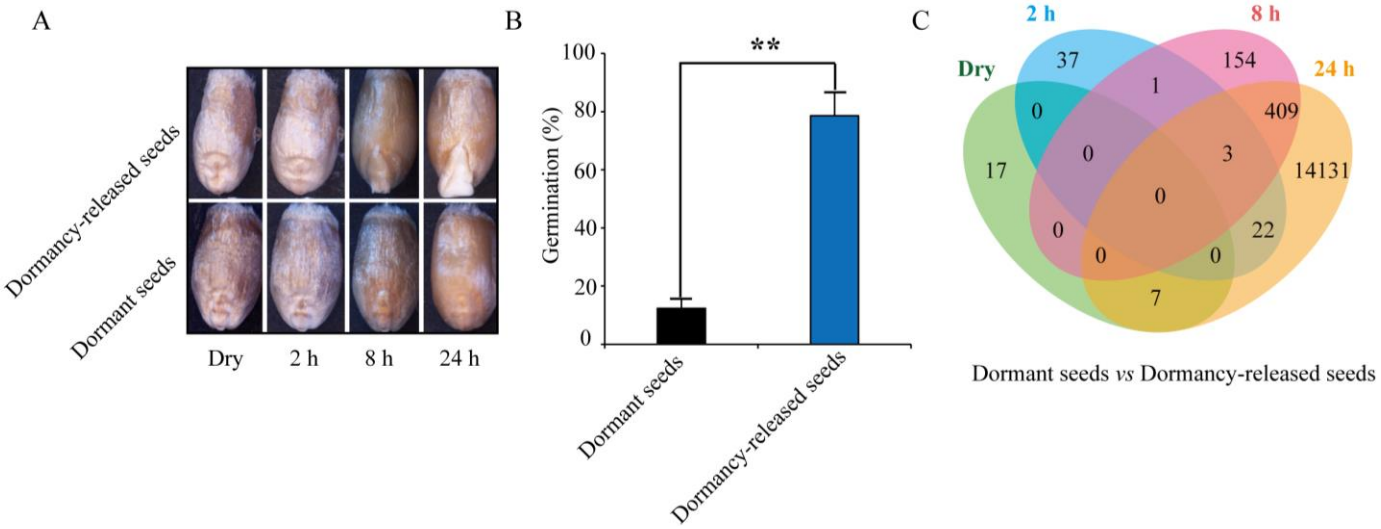

Fig. S19 RNA-seq analysis of dormant seeds and dormancy-released seeds of wheat landrace Darius. (A) Different germination process of dormant seeds and dormancy-released seeds of Darius. (B) Germination phenotypic of dormant seeds and dormancy-released seeds of Darius. Significant differences were determined by Student's *t*-test. *, $0.01<P<0.05$; **, $P<0.01$. (C) Venn diagrams showing differentially expressed genes between dormant seeds and dormancy-released seeds at different imbibition stage.
